# Supplementary figures and images for: MDGAs are fast-diffusing molecules that delay excitatory synapse development by altering neuroligin behavior
Source: eLife. 2022 May 9;11:e75233. doi: 10.7554/eLife.75233 (PMC9084894; doi:10.7554/eLife.75233)

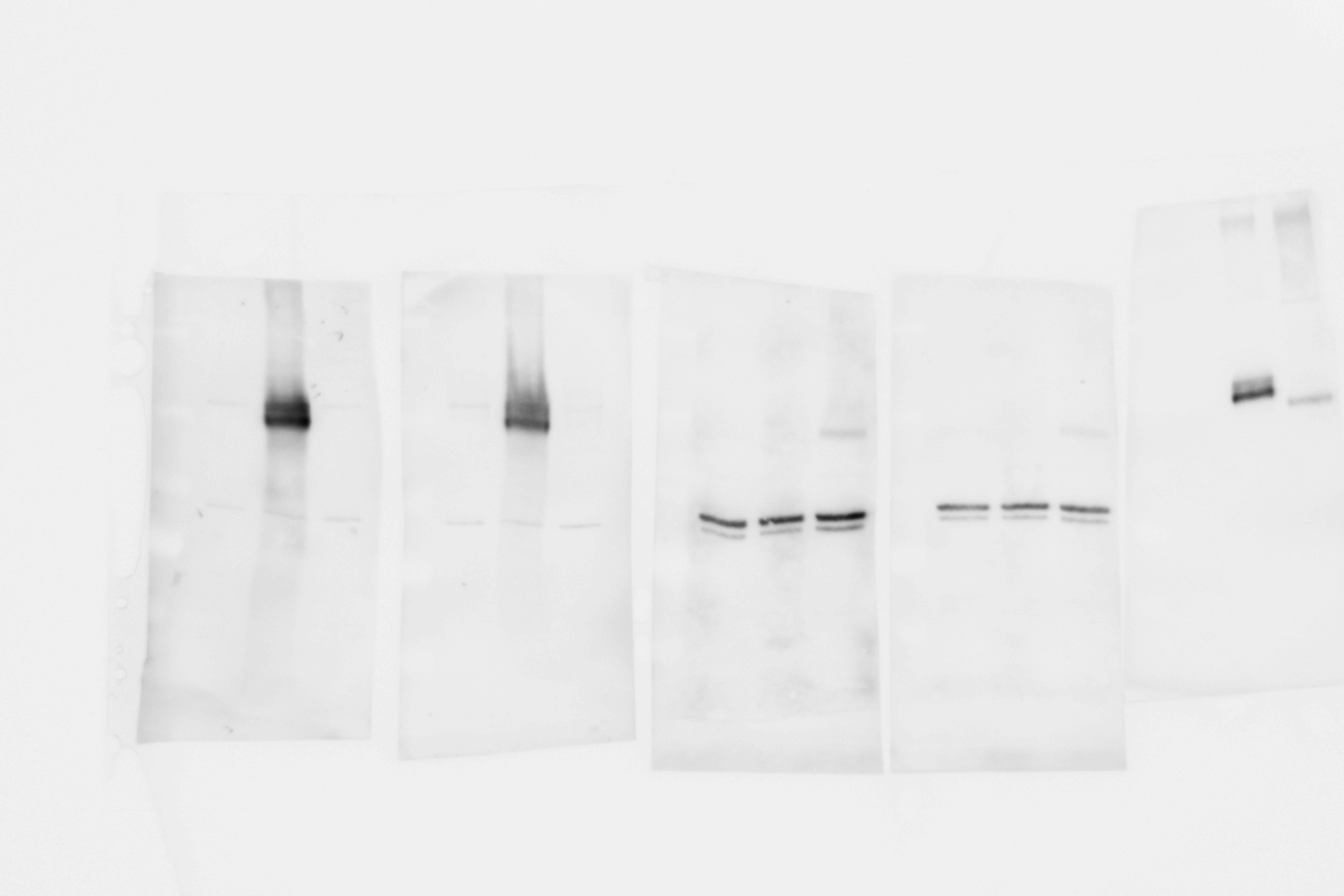

Supplement: Figure 1—source data 1. [file elife-75233-fig1-data1.zip › Figure 1 ΓÇô source data 1.tif]

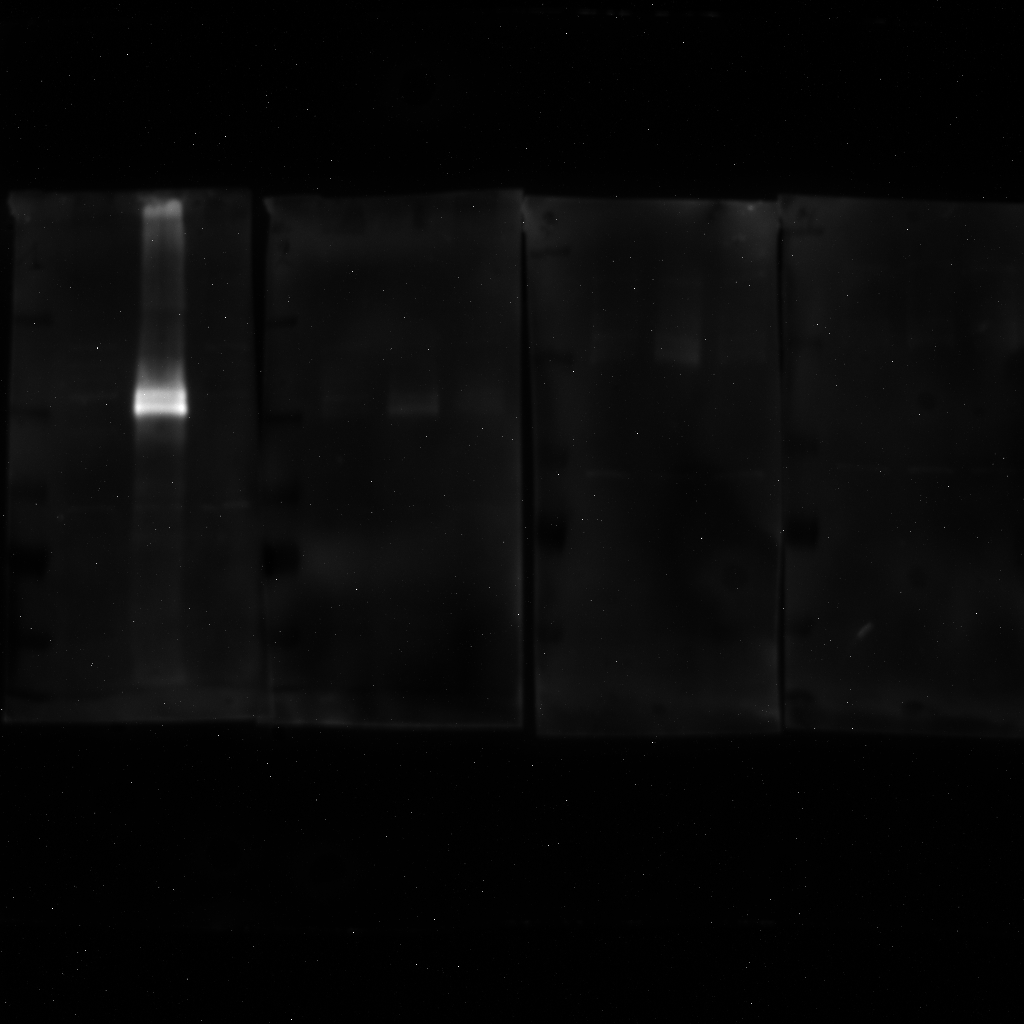

Supplement: Figure 1—source data 2. [file elife-75233-fig1-data2.zip › Figure 1 ΓÇô source data 2.TIF]

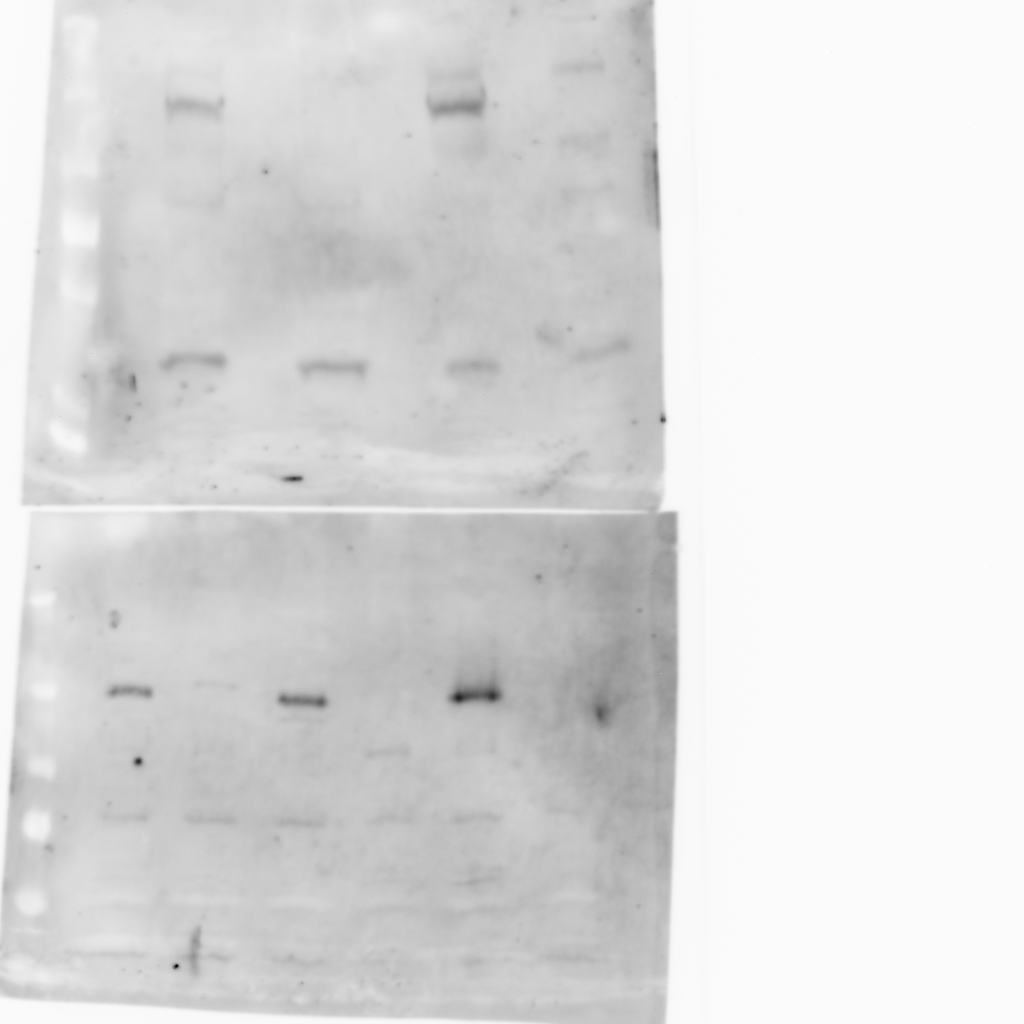

Supplement: Figure 1—source data 3. [file elife-75233-fig1-data3.zip › Figure 1 ΓÇô source data 3.tif]

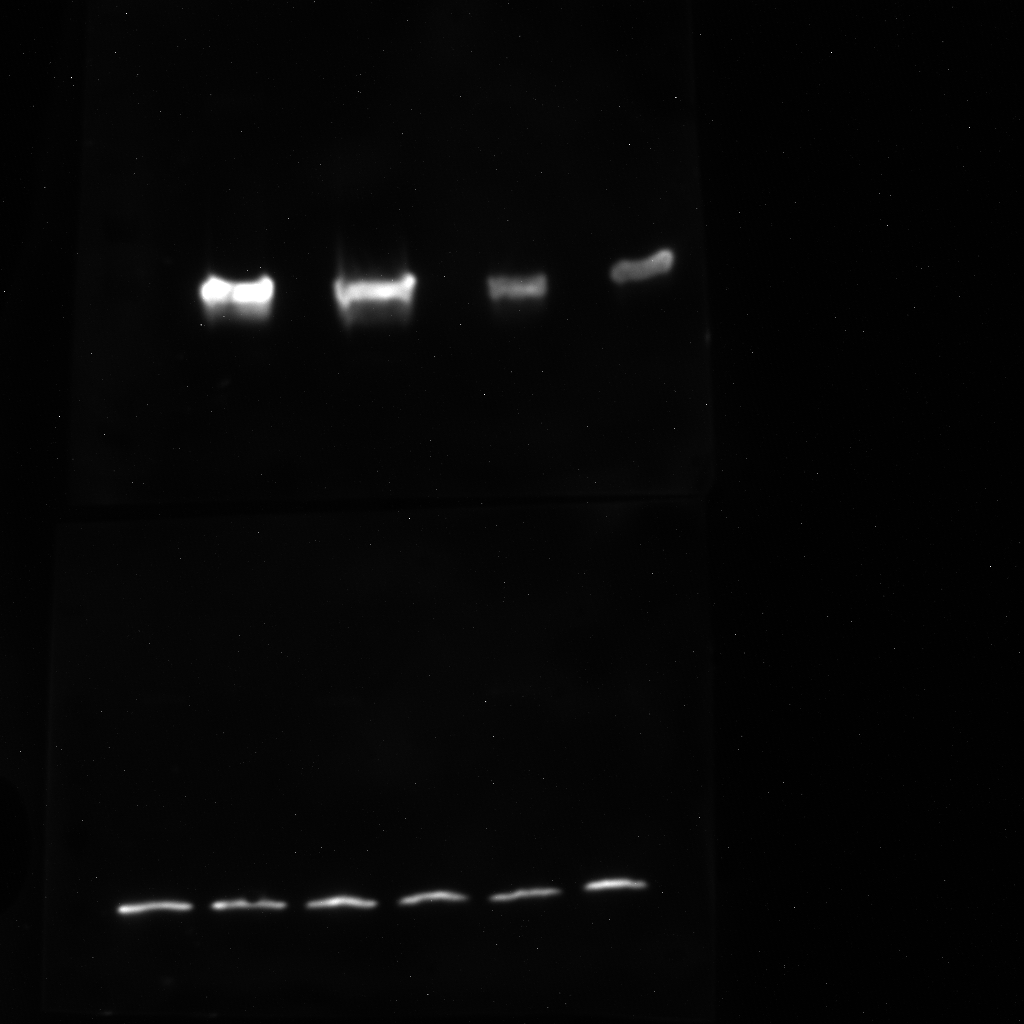

Supplement: Figure 1—source data 4. [file elife-75233-fig1-data4.zip › Figure 1 ΓÇô source data 4.TIF]

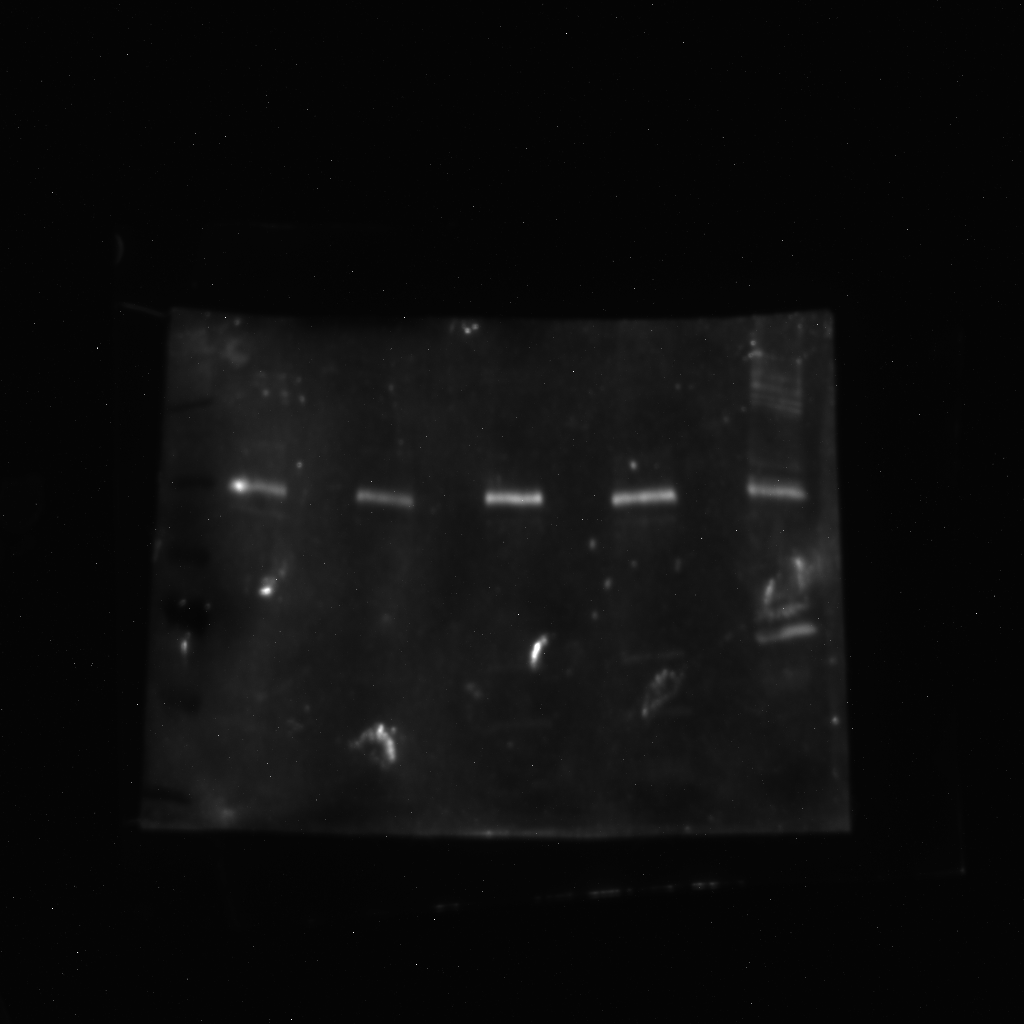

Supplement: Figure 1—source data 5. [file elife-75233-fig1-data5.zip › Figure 1 ΓÇô source data 5.TIF]

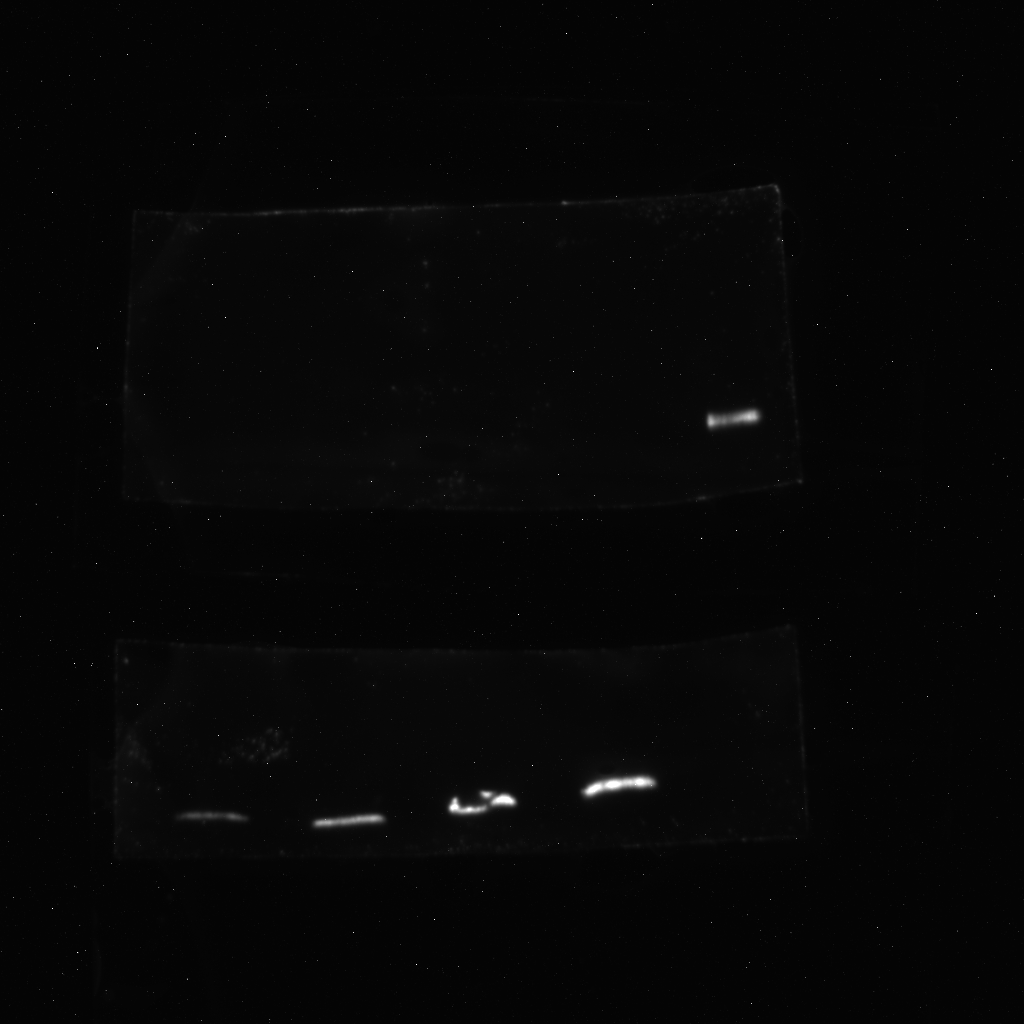

Supplement: Figure 1—source data 6. [file elife-75233-fig1-data6.zip › Figure 1 ΓÇô source data 6.TIF]

**Figure 1A**

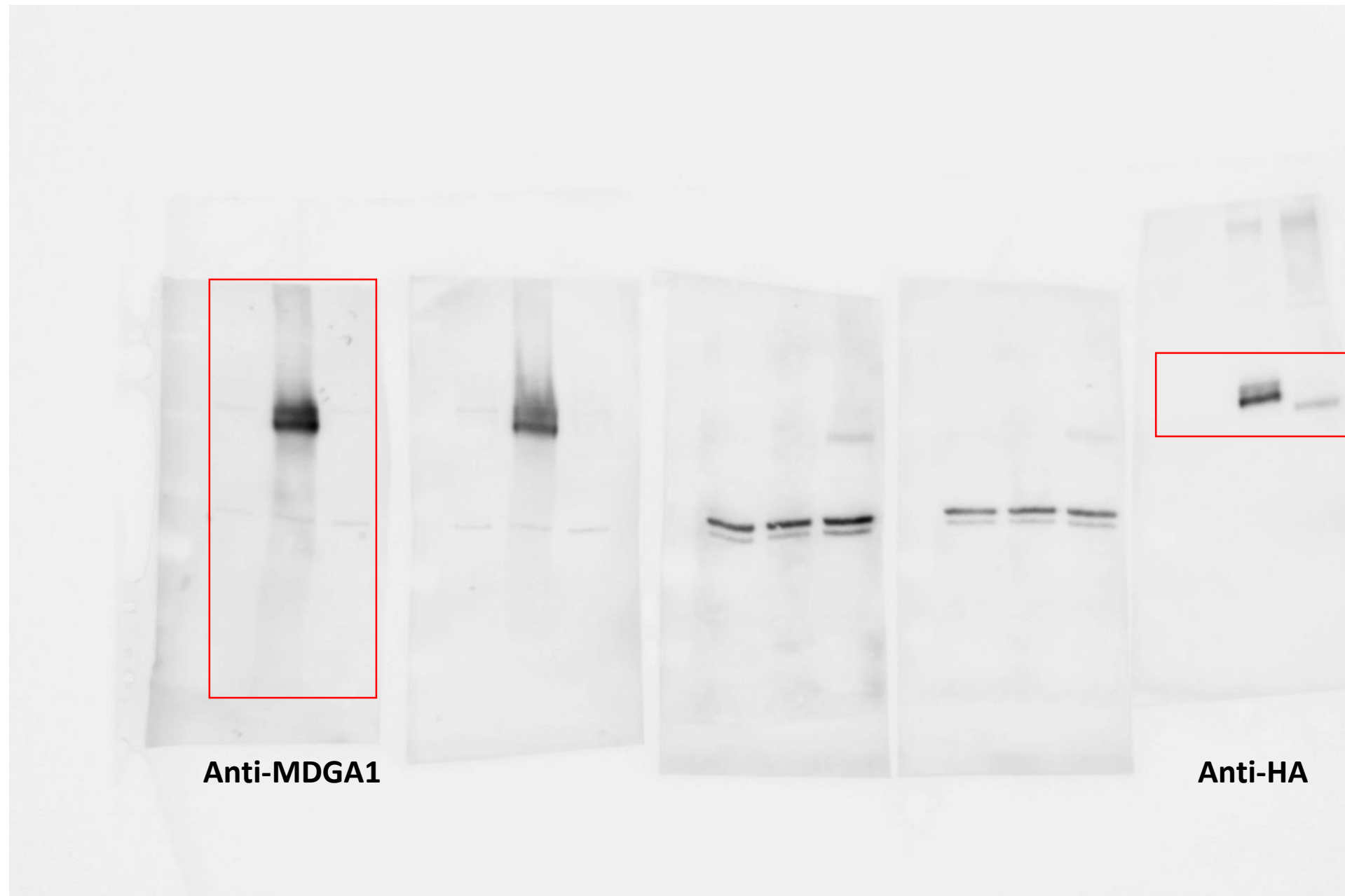

**Figure 1B**

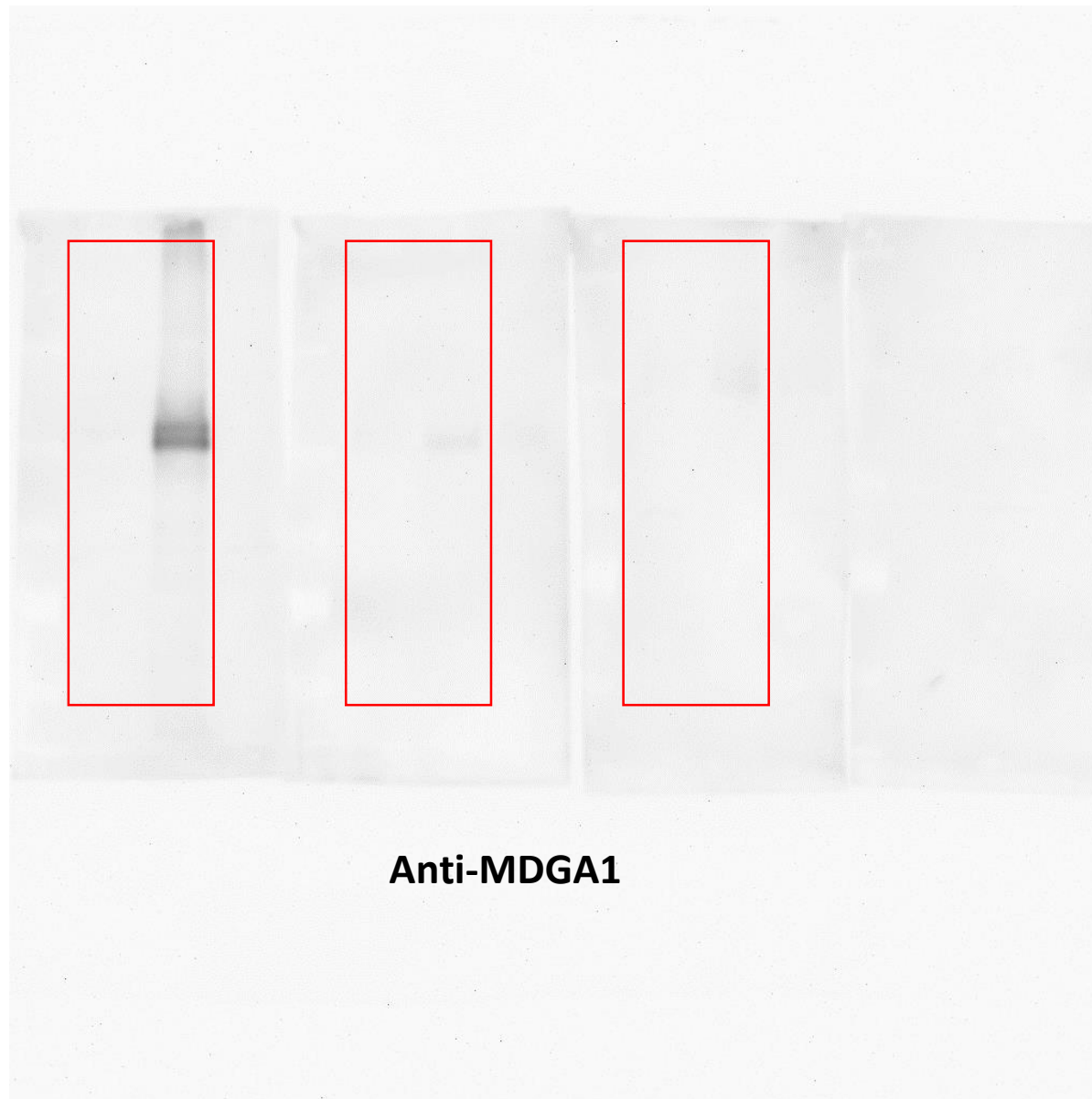

**Figure 1C**

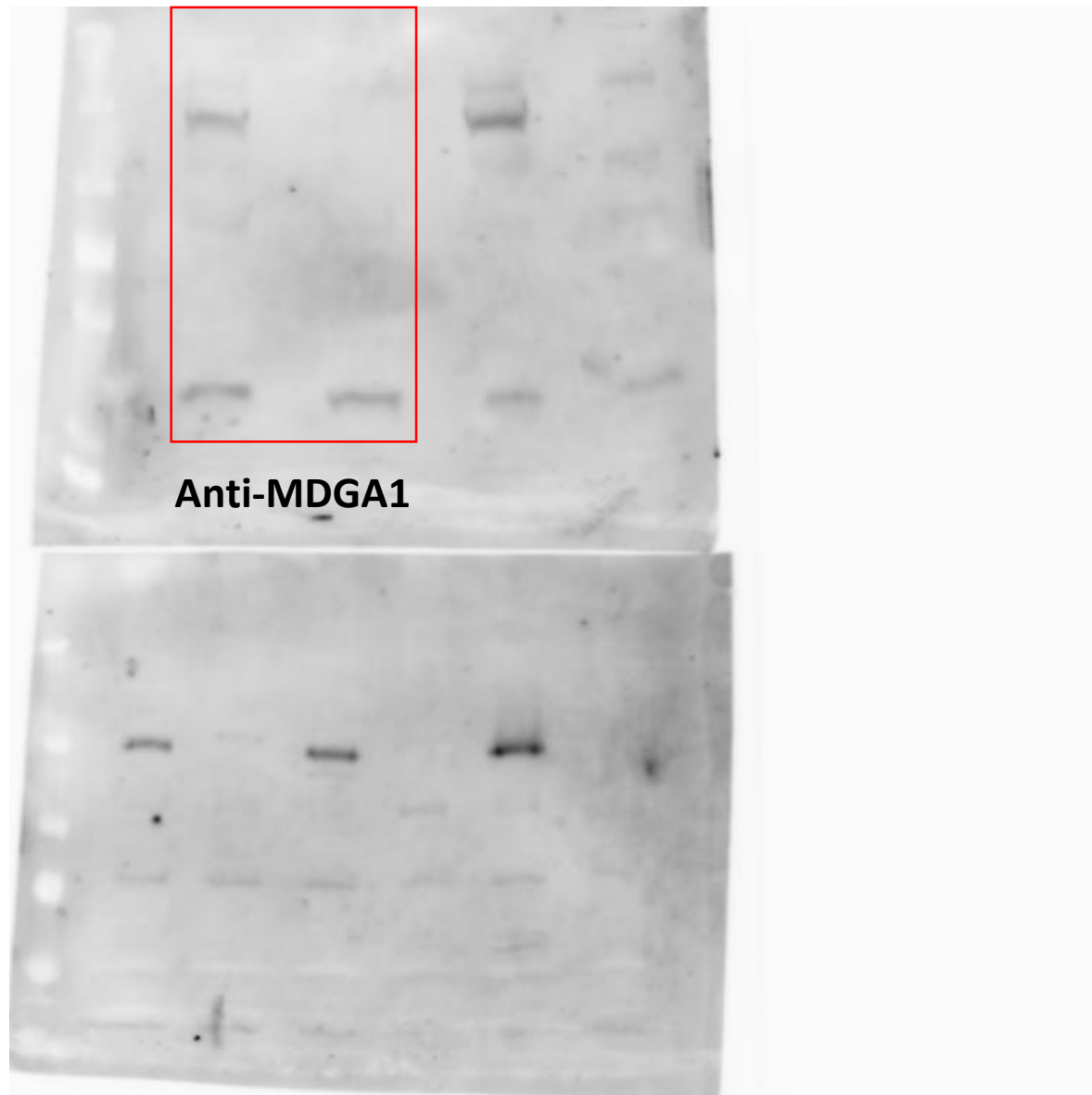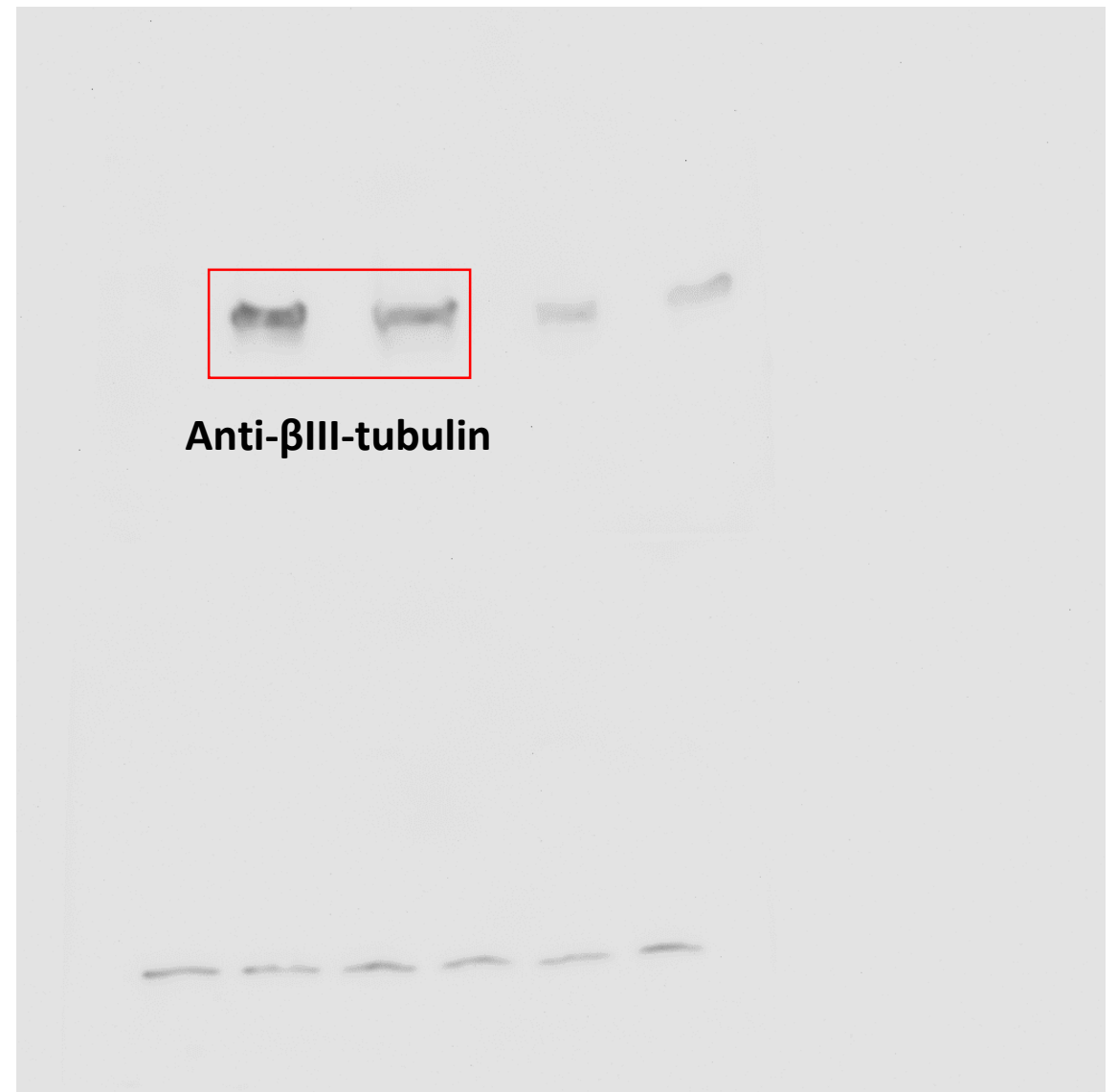

**Figure 1E**

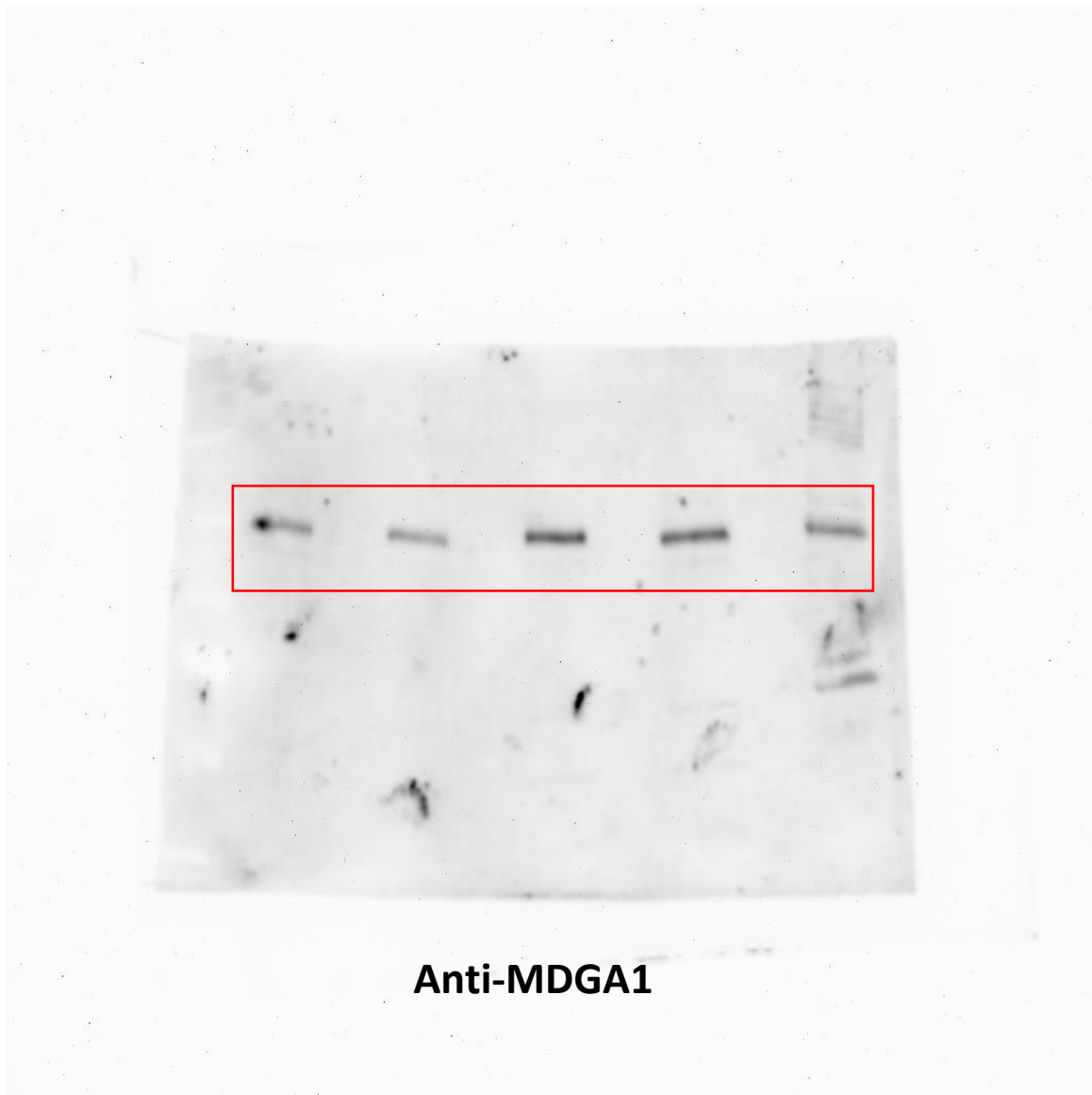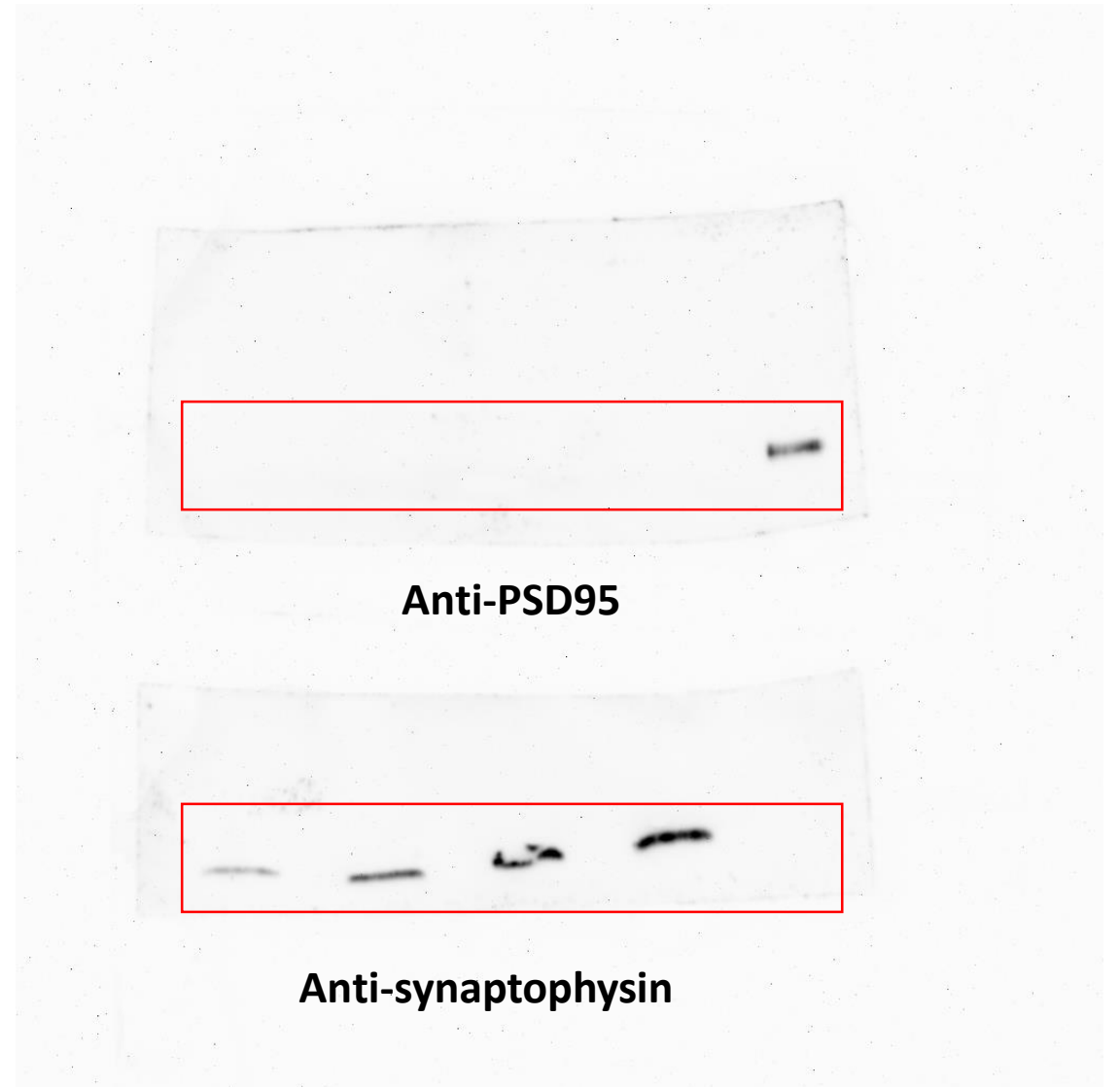

Supplement: Figure 1—source data 7. [file elife-75233-fig1-data7.pdf]

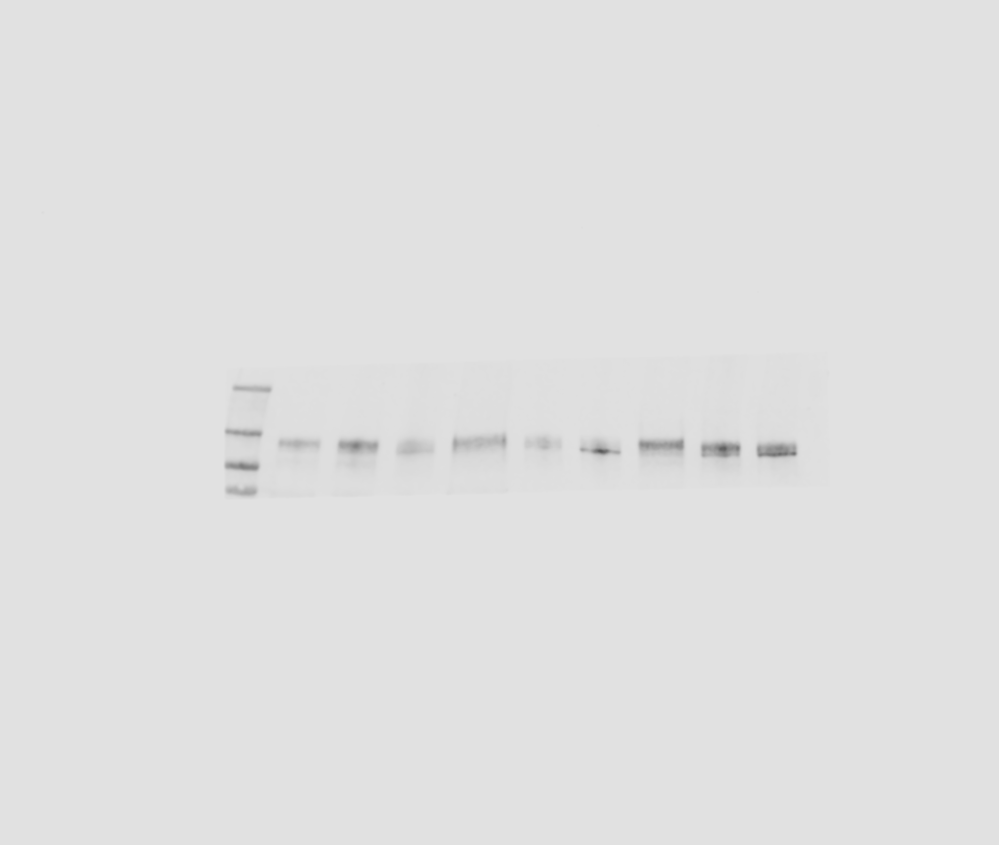

Supplement: Figure 1—figure supplement 1—source data 1. [file elife-75233-fig1-figsupp1-data1.zip › Figure 1 ΓÇô figure supplement 1 ΓÇô source data 1.tif]

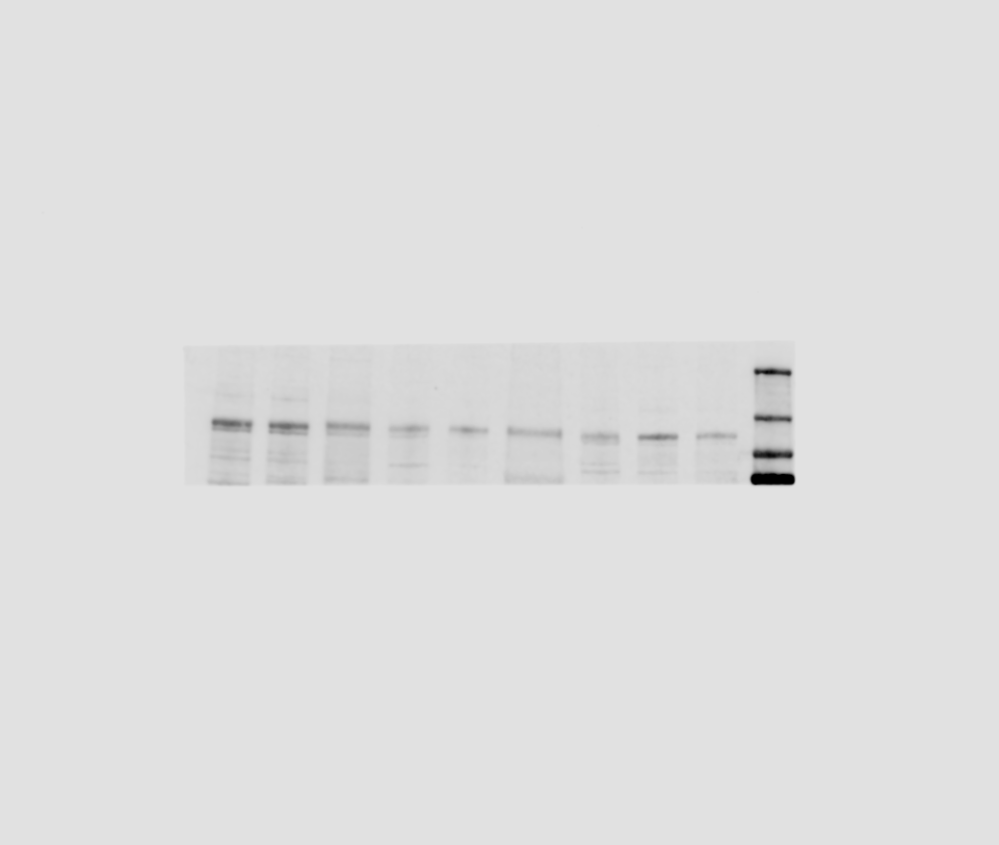

Supplement: Figure 1—figure supplement 1—source data 2. [file elife-75233-fig1-figsupp1-data2.zip › Figure 1 ΓÇô figure supplement 1 ΓÇô source data 2.tif]

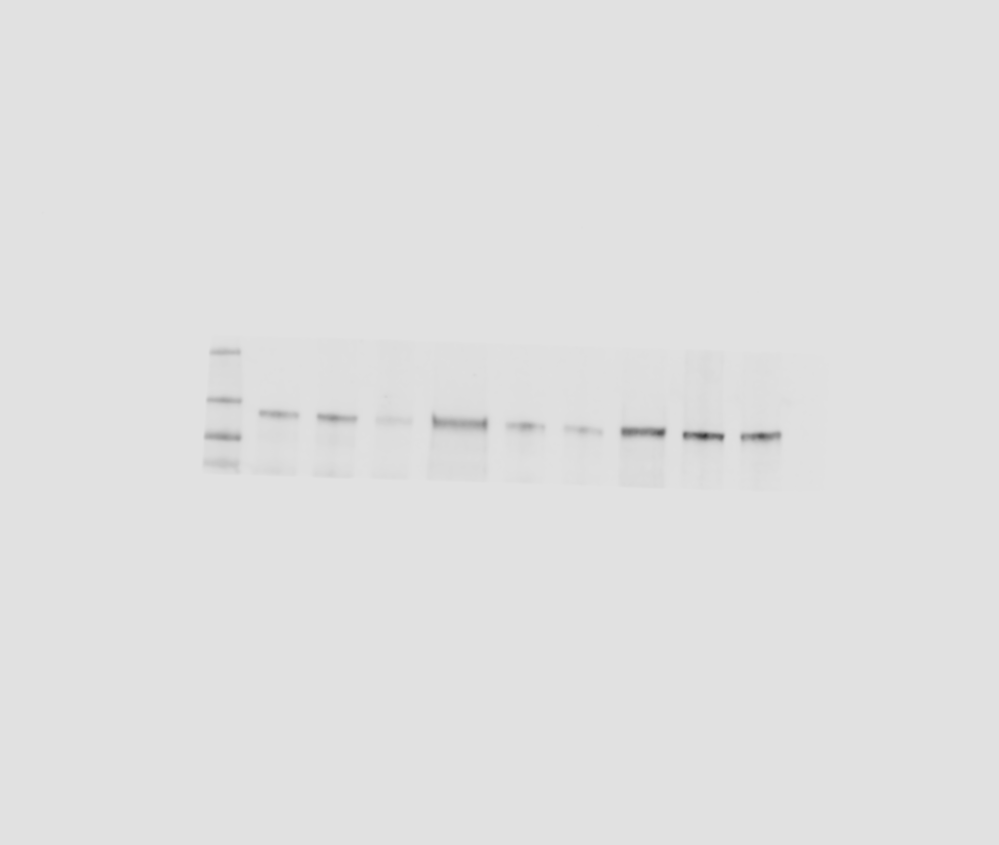

Supplement: Figure 1—figure supplement 1—source data 3. [file elife-75233-fig1-figsupp1-data3.zip › Figure 1 ΓÇô figure supplement 1 ΓÇô source data 3.tif]

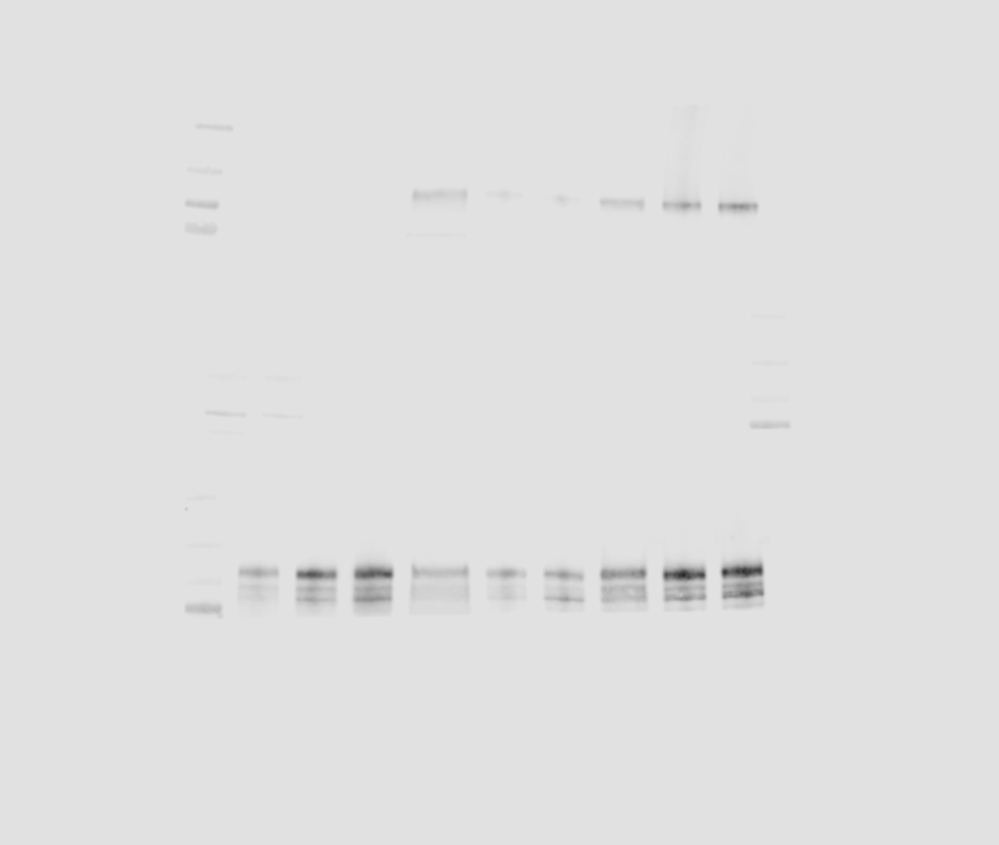

Supplement: Figure 1—figure supplement 1—source data 4. [file elife-75233-fig1-figsupp1-data4.zip › Figure 1 ΓÇô figure supplement 1 ΓÇô source data 4.tif]

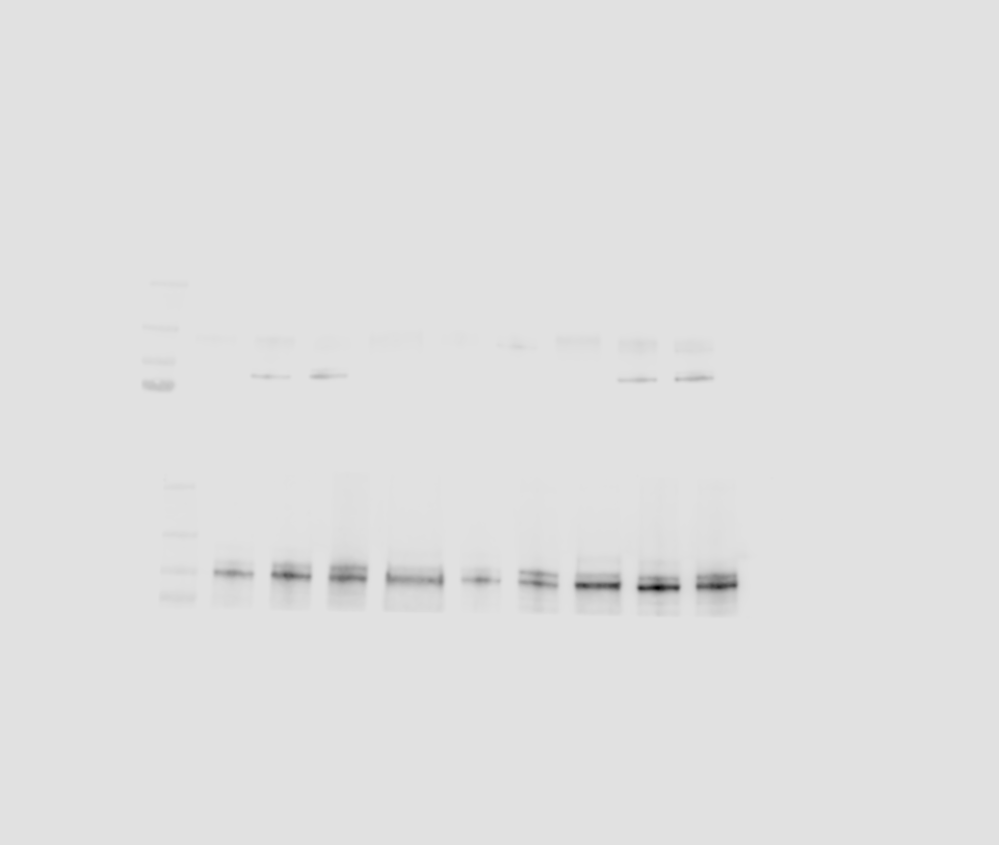

Supplement: Figure 1—figure supplement 1—source data 5. [file elife-75233-fig1-figsupp1-data5.zip › Figure 1 ΓÇô figure supplement 1 ΓÇô source data 5.tif]

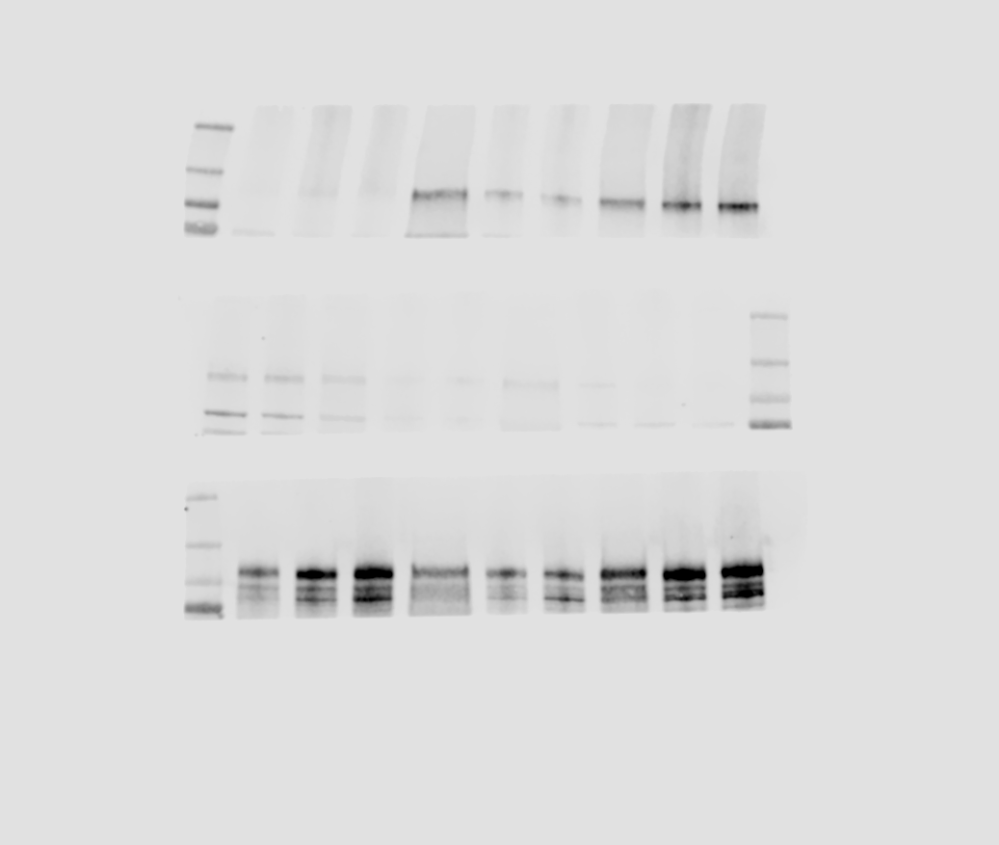

Supplement: Figure 1—figure supplement 1—source data 6. [file elife-75233-fig1-figsupp1-data6.zip › Figure 1 ΓÇô figure supplement 1 ΓÇô source data 6.tif]

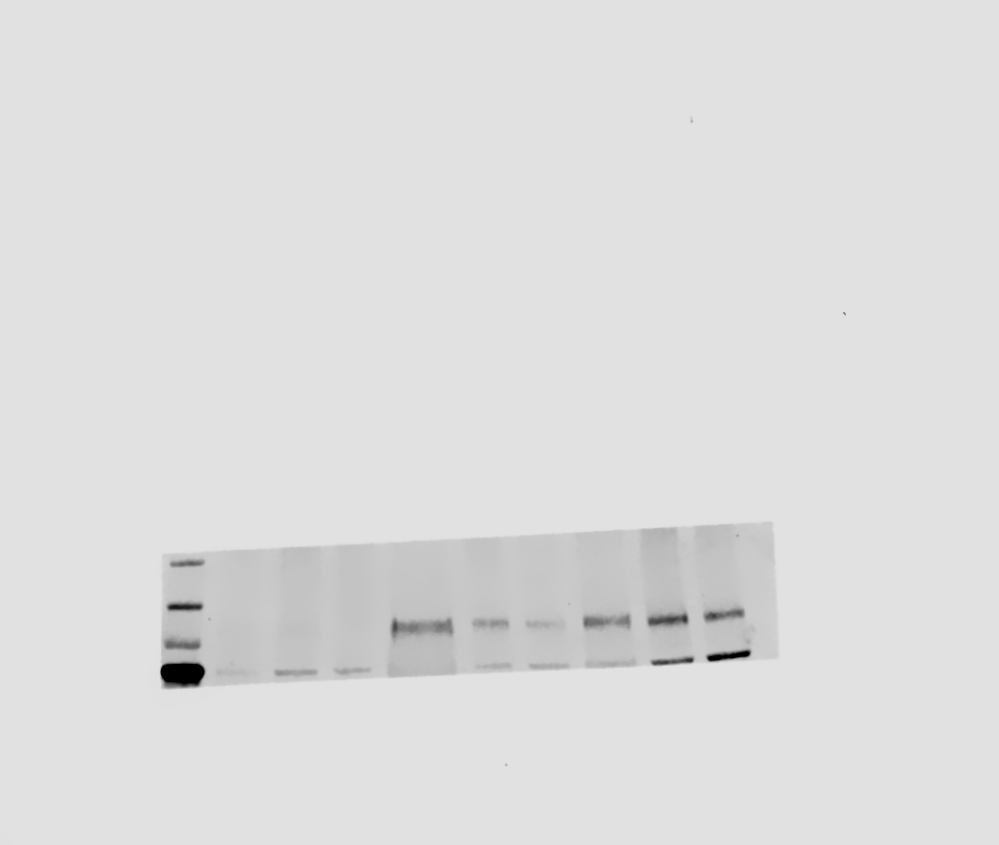

Supplement: Figure 1—figure supplement 1—source data 7. [file elife-75233-fig1-figsupp1-data7.zip › Figure 1 ΓÇô figure supplement 1 ΓÇô source data 7.tif]

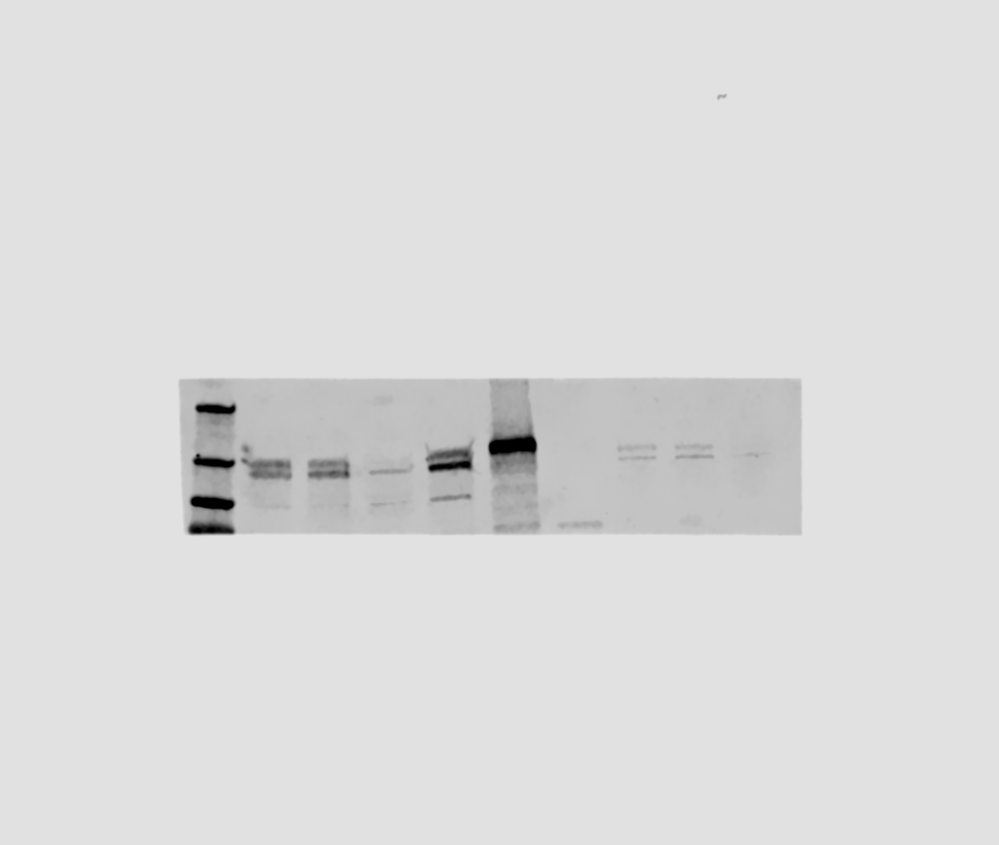

Supplement: Figure 1—figure supplement 1—source data 8. [file elife-75233-fig1-figsupp1-data8.zip › Figure 1 ΓÇô figure supplement 1 ΓÇô source data 8.tif]

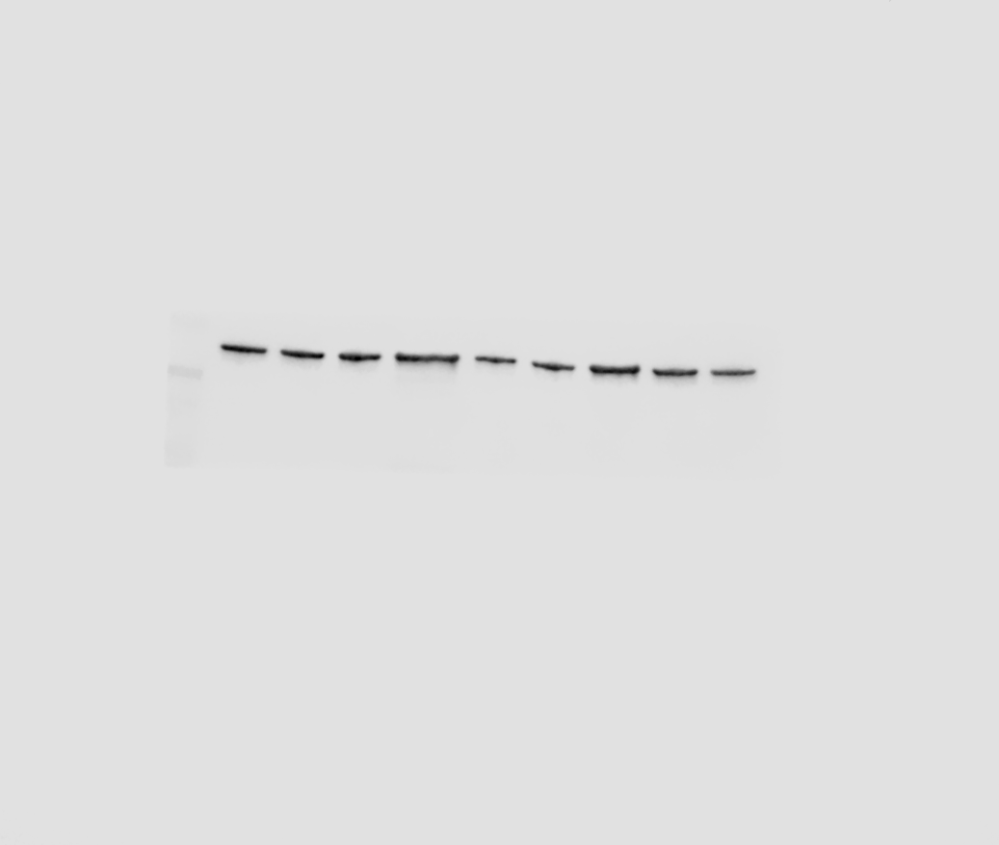

Supplement: Figure 1—figure supplement 1—source data 9. [file elife-75233-fig1-figsupp1-data9.zip › Figure 1 ΓÇô figure supplement 1 ΓÇô source data 9.tif]

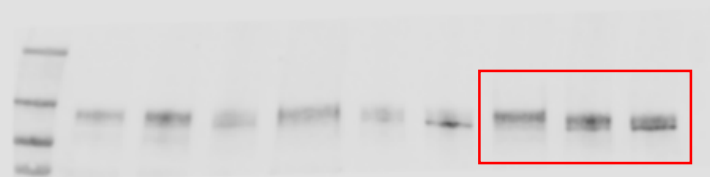

**Anti-NLGN1**

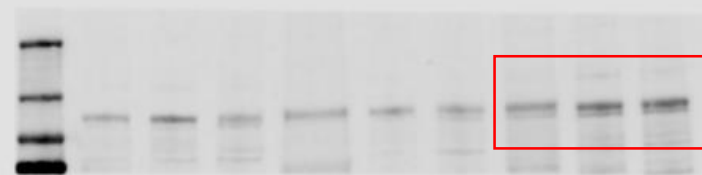

**Anti-NLGN2**

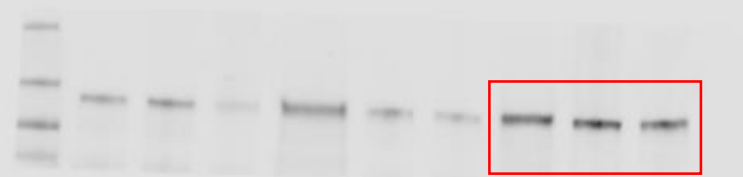

**Anti-NLGN3**

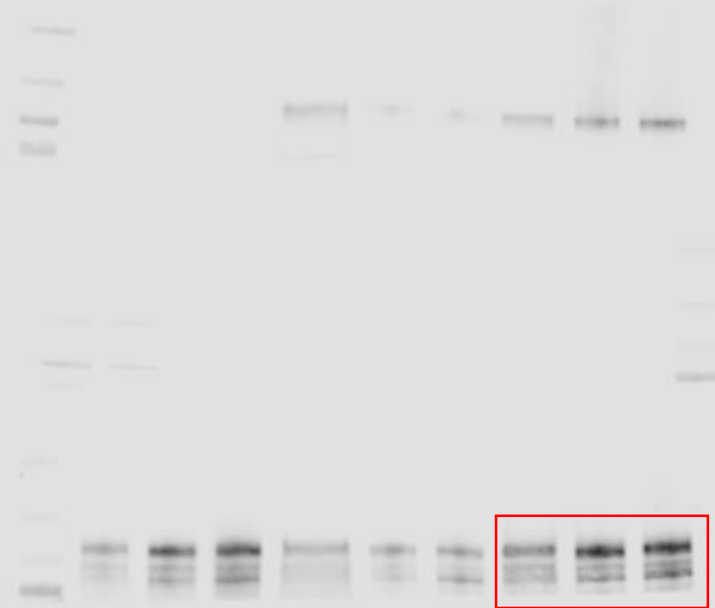

**Anti-PSD-95**

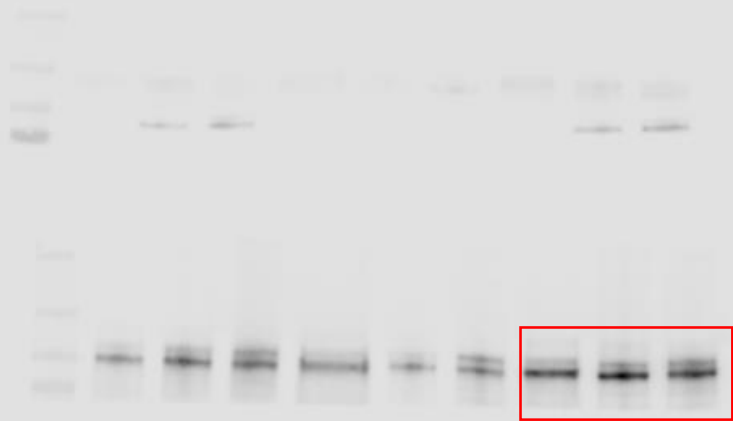

**Anti-Gephyrin**

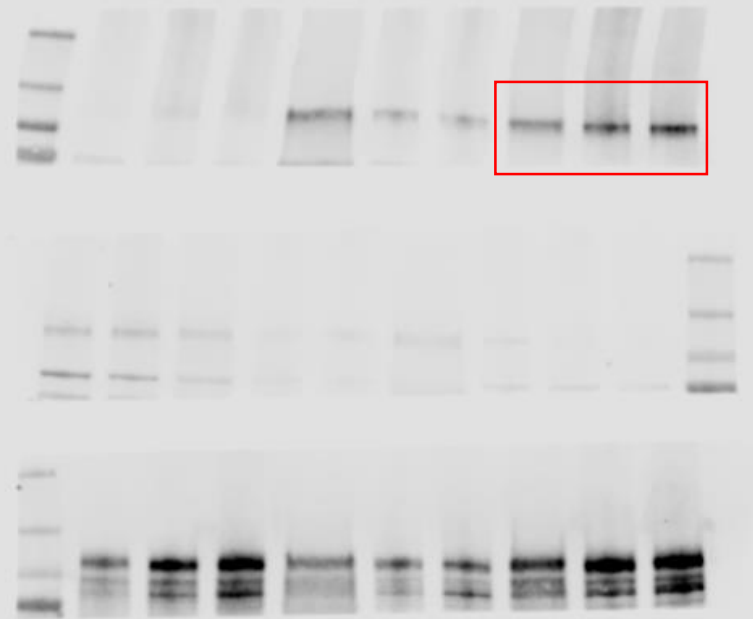

**Anti-GluA1**

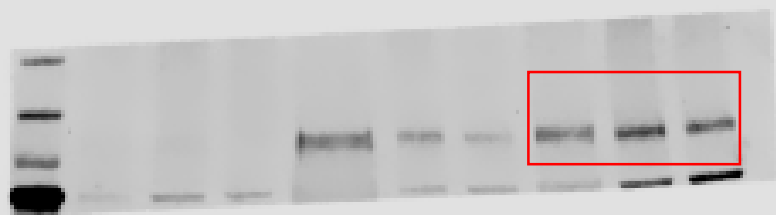

**Anti-GluA2**

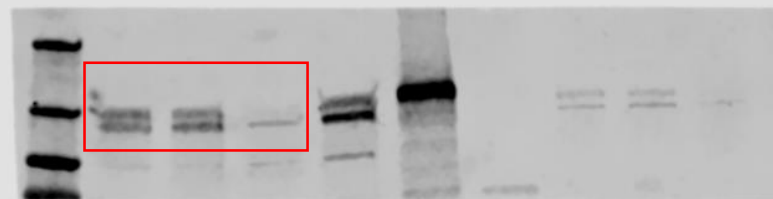

**Anti-MDGA1**

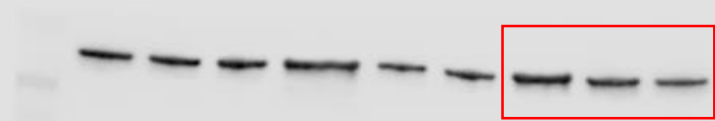

**Anti-Actin**

Supplement: Figure 1—figure supplement 1—source data 10. [file elife-75233-fig1-figsupp1-data10.pdf]

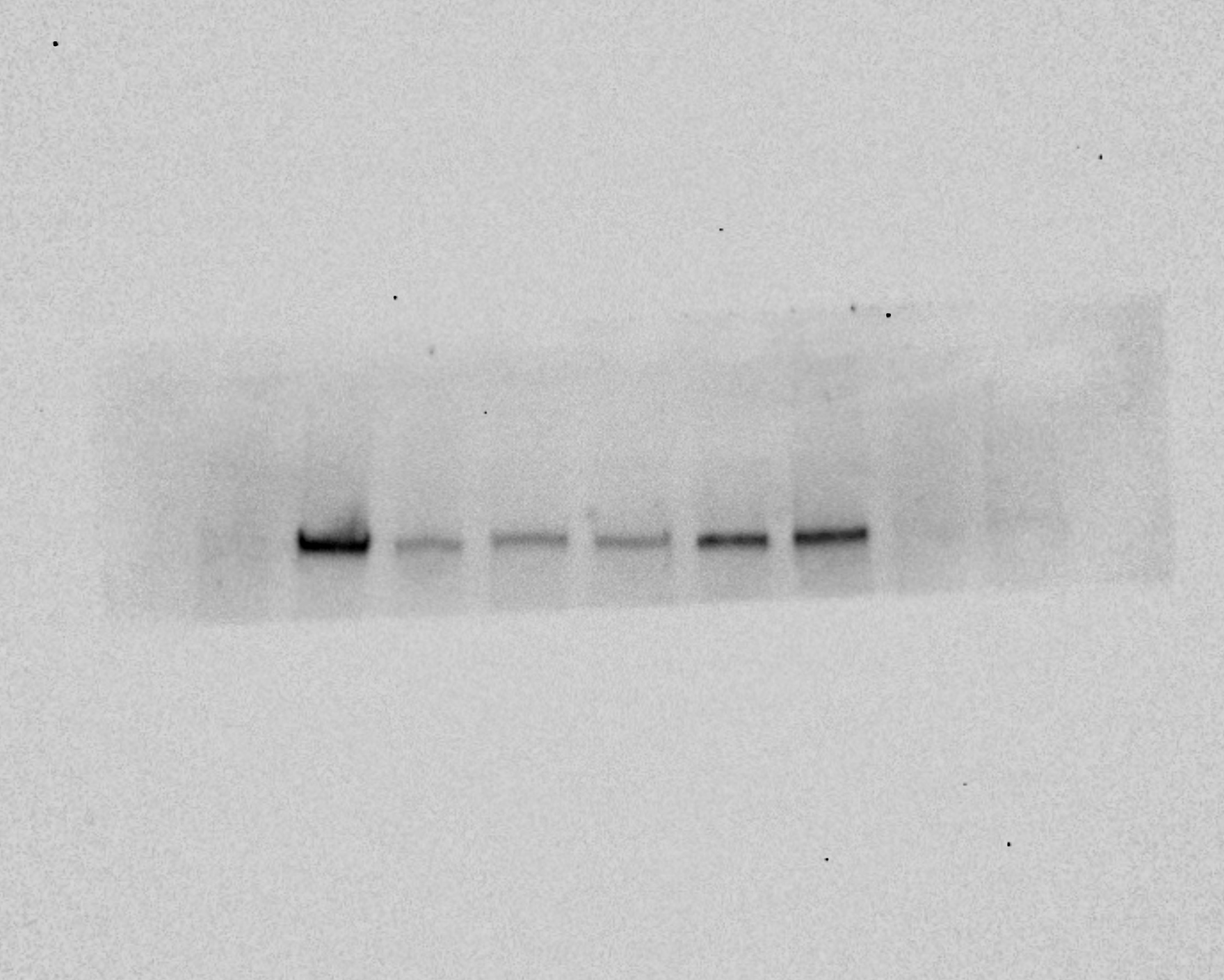

Supplement: Figure 2—figure supplement 1—source data 1. [file elife-75233-fig2-figsupp1-data1.zip › Figure 2 ΓÇô figure supplement 1 ΓÇô source data 1.jpg]

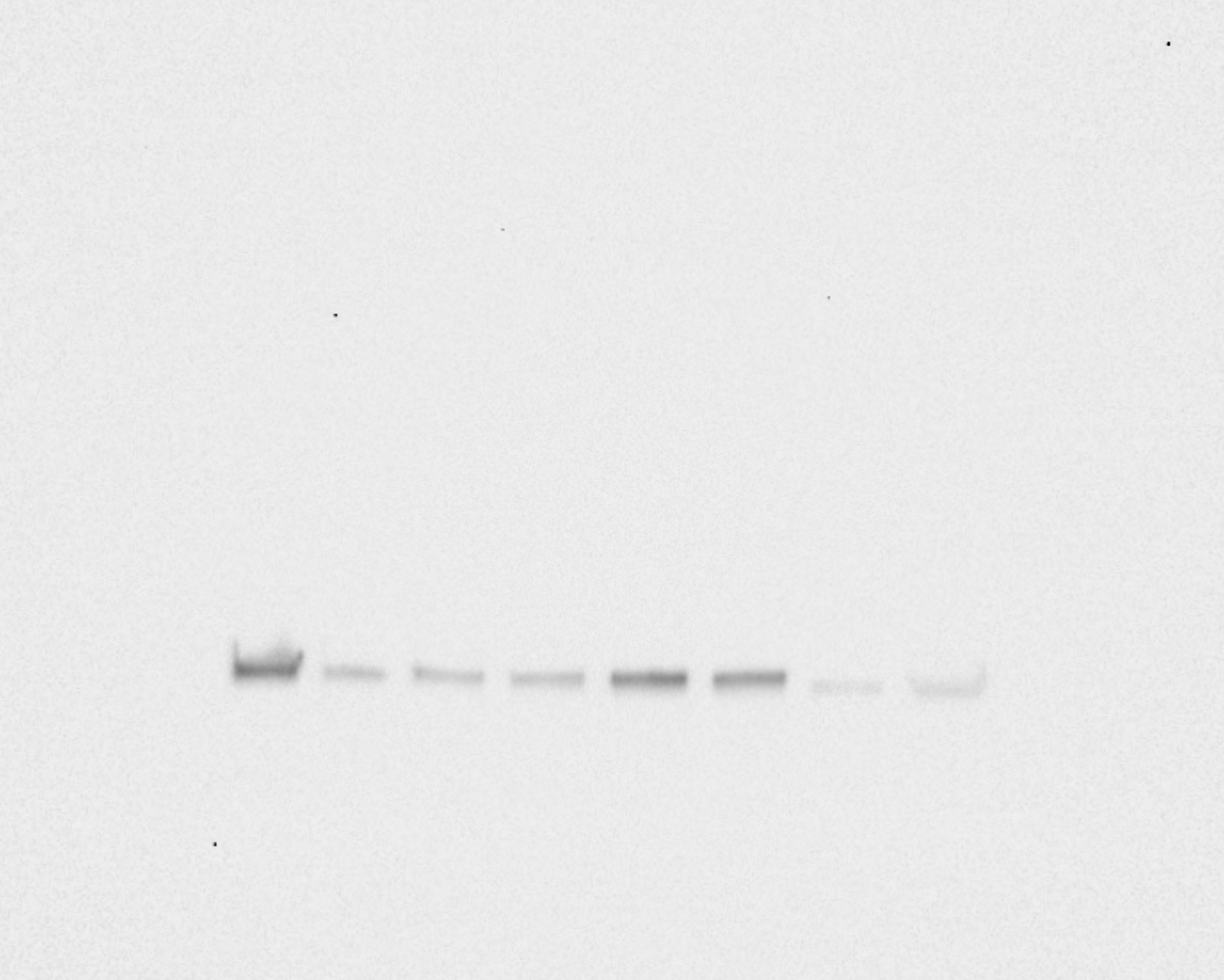

Supplement: Figure 2—figure supplement 1—source data 2. [file elife-75233-fig2-figsupp1-data2.zip › Figure 2 ΓÇô figure supplement 1 ΓÇô source data 2.jpg]

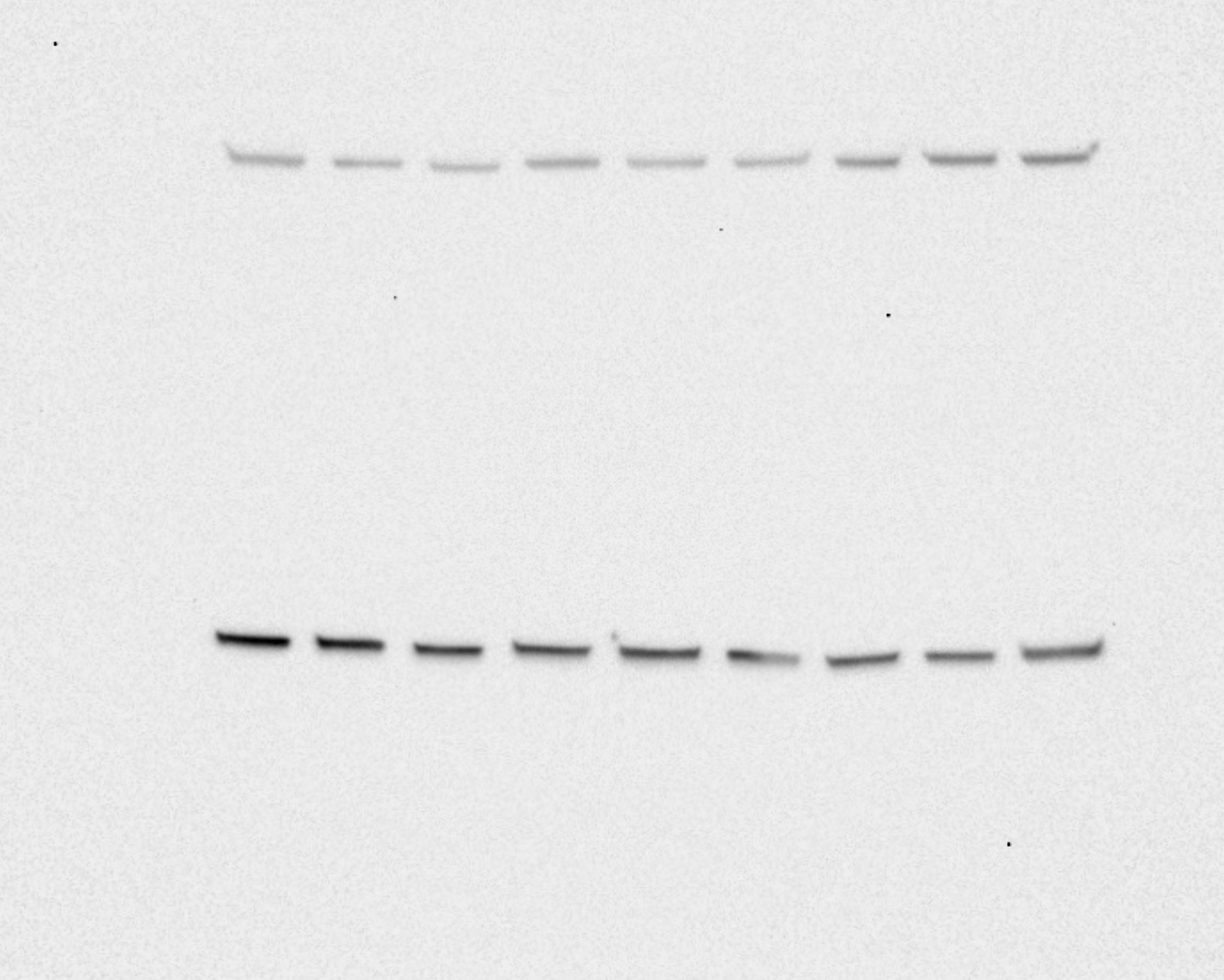

Supplement: Figure 2—figure supplement 1—source data 3. [file elife-75233-fig2-figsupp1-data3.zip › Figure 2 ΓÇô figure supplement 1 ΓÇô source data 3.jpg]

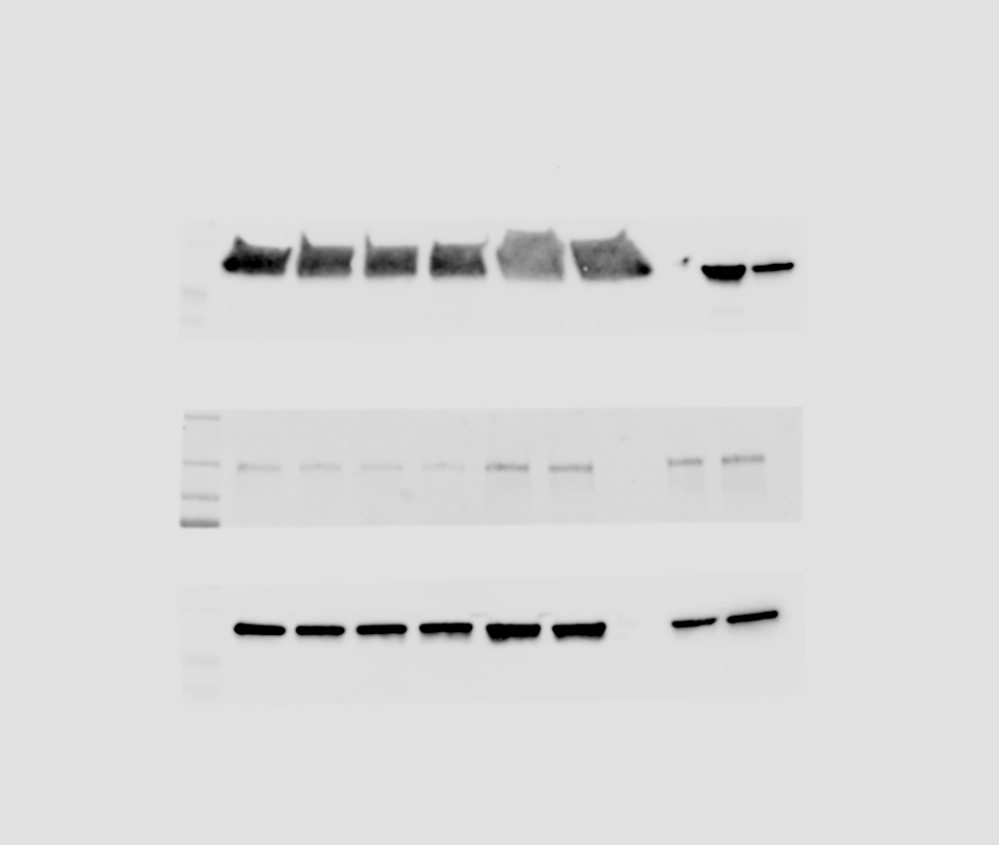

Supplement: Figure 2—figure supplement 1—source data 4. [file elife-75233-fig2-figsupp1-data4.zip › Figure 2 ΓÇô figure supplement 1 ΓÇô source data 4.png]

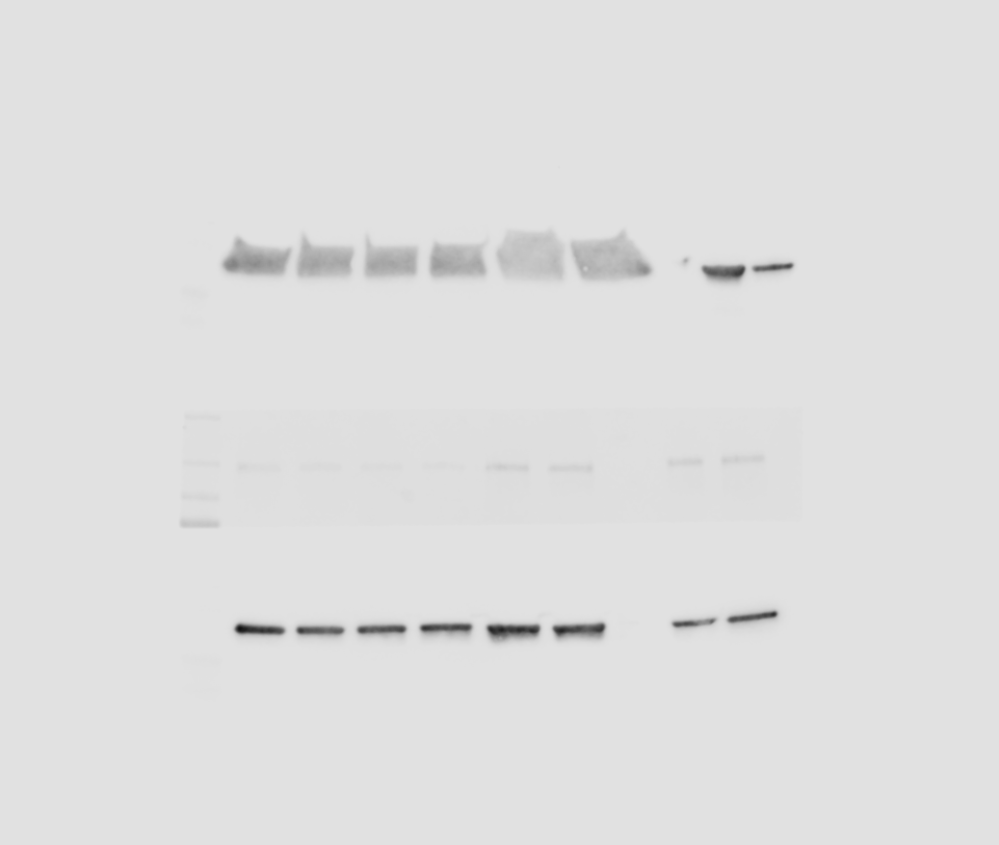

Supplement: Figure 2—figure supplement 1—source data 5. [file elife-75233-fig2-figsupp1-data5.zip › Figure 2 ΓÇô figure supplement 1 ΓÇô source data 5.png]

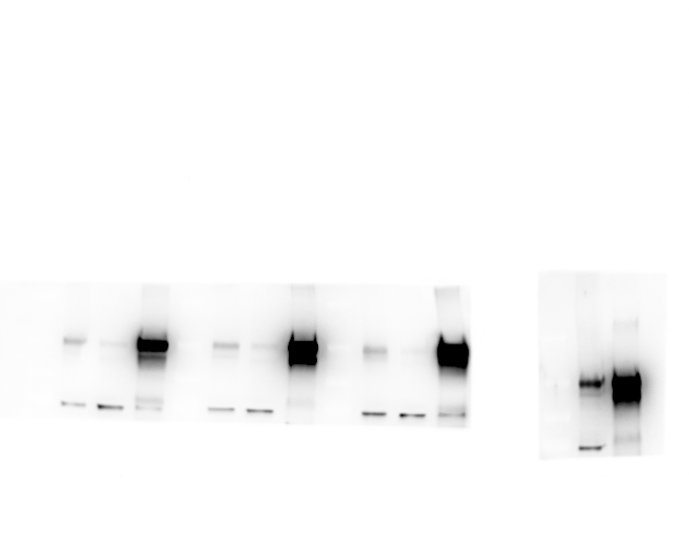

Supplement: Figure 2—figure supplement 3—source data 1. [file elife-75233-fig2-figsupp3-data1.zip › Figure 2 ΓÇô figure supplement 3 ΓÇô source data 1.jpg]

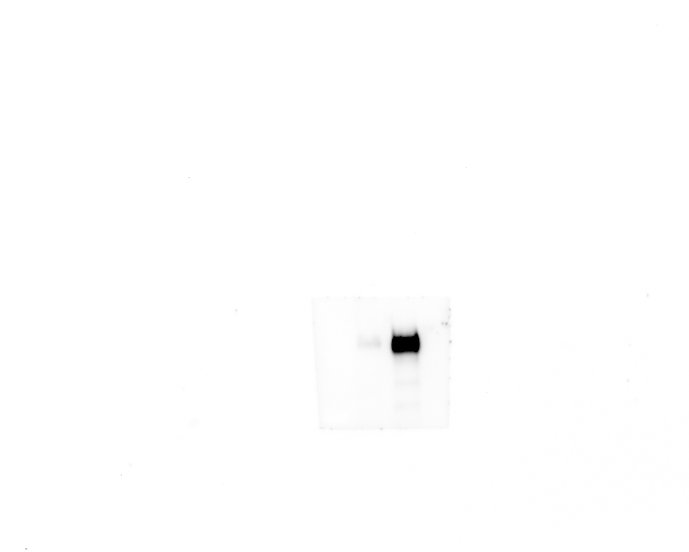

Supplement: Figure 2—figure supplement 3—source data 2. [file elife-75233-fig2-figsupp3-data2.zip › Figure 2 ΓÇô figure supplement 3 ΓÇô source data 2.jpg]

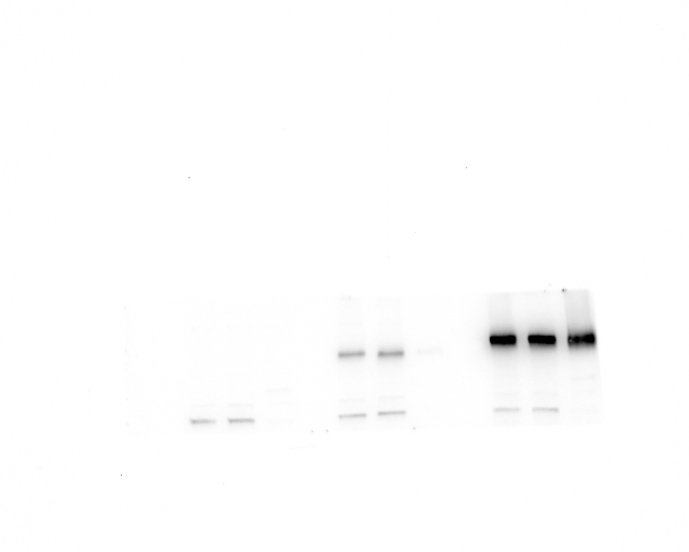

Supplement: Figure 2—figure supplement 3—source data 4. [file elife-75233-fig2-figsupp3-data4.zip › Figure 2 ΓÇô figure supplement 3 ΓÇô source data 4.jpg]

**Figure 2 – figure supplement 3A**

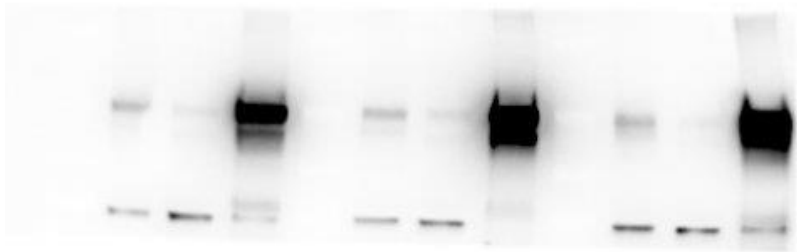

**Anti-NLGN1**

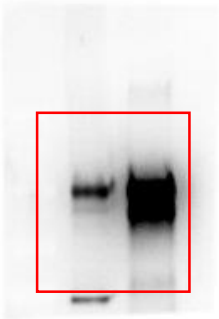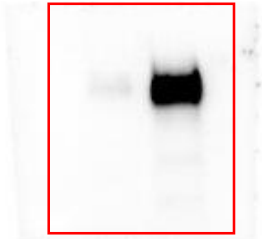

**Anti-MDGA1**

**Figure 2 – figure supplement 3B**

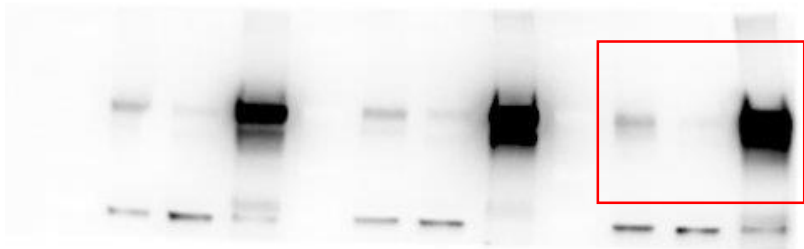

**Anti-NLGN1**

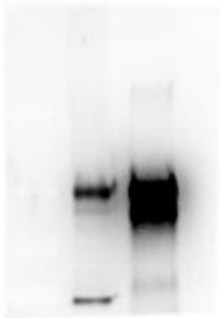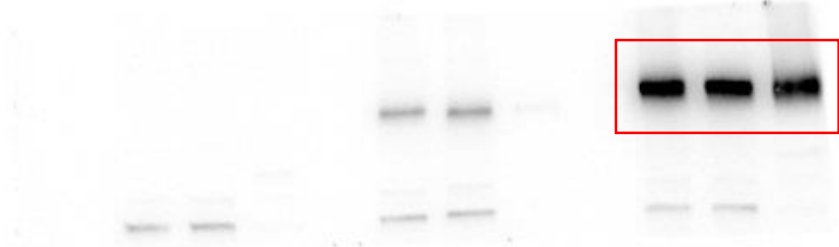

**Anti-HA**

Supplement: Figure 2—figure supplement 3—source data 5. [file elife-75233-fig2-figsupp3-data5.pdf]

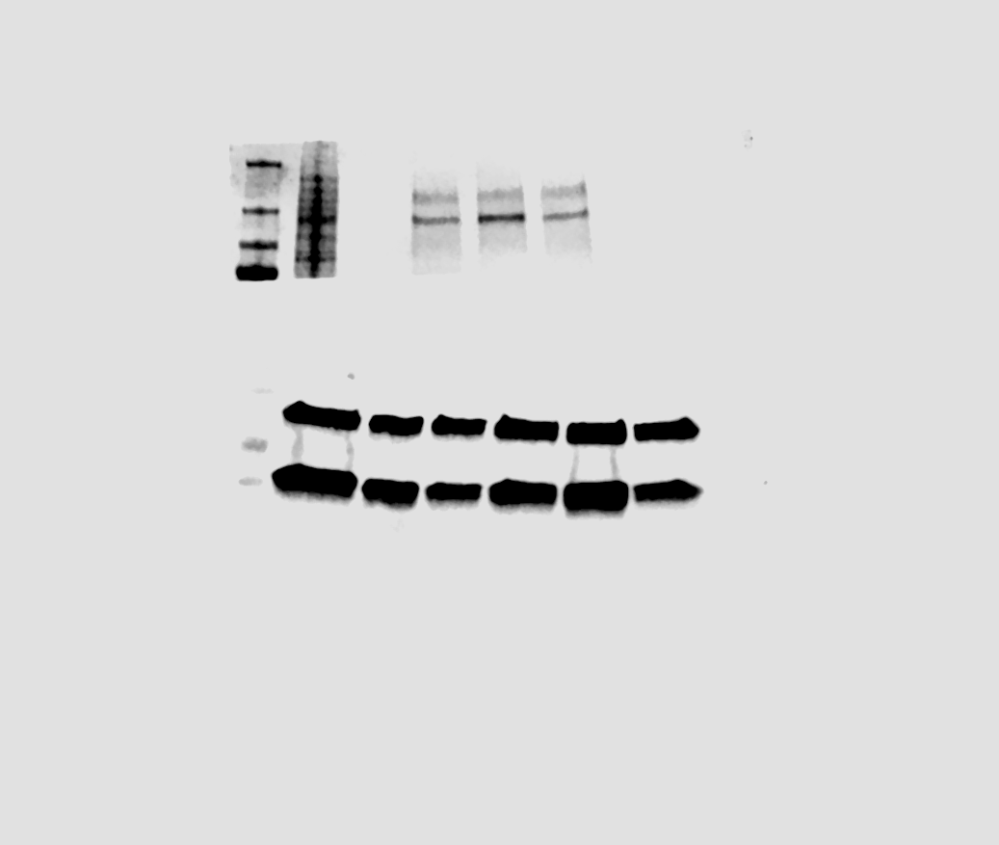

Supplement: Figure 6—source data 1. [file elife-75233-fig6-data1.zip › Figure 6 ΓÇô source data 1.tif]

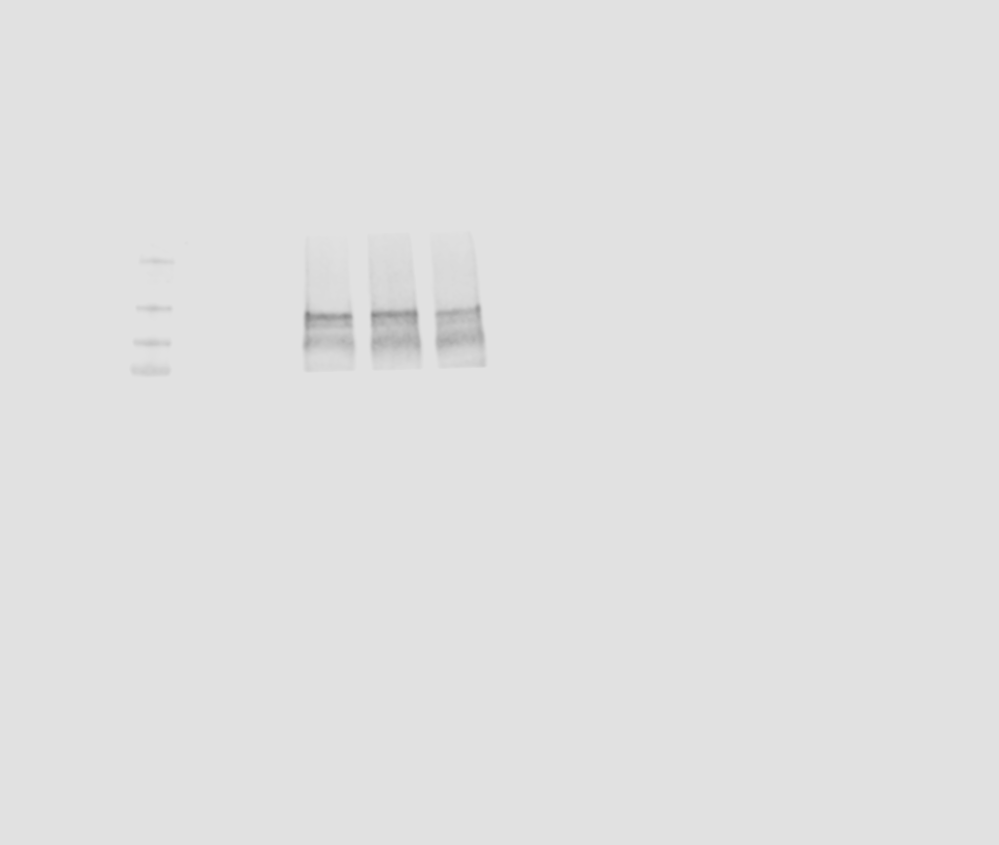

Supplement: Figure 6—source data 2. [file elife-75233-fig6-data2.zip › Figure 6 ΓÇô source data 2.tif]

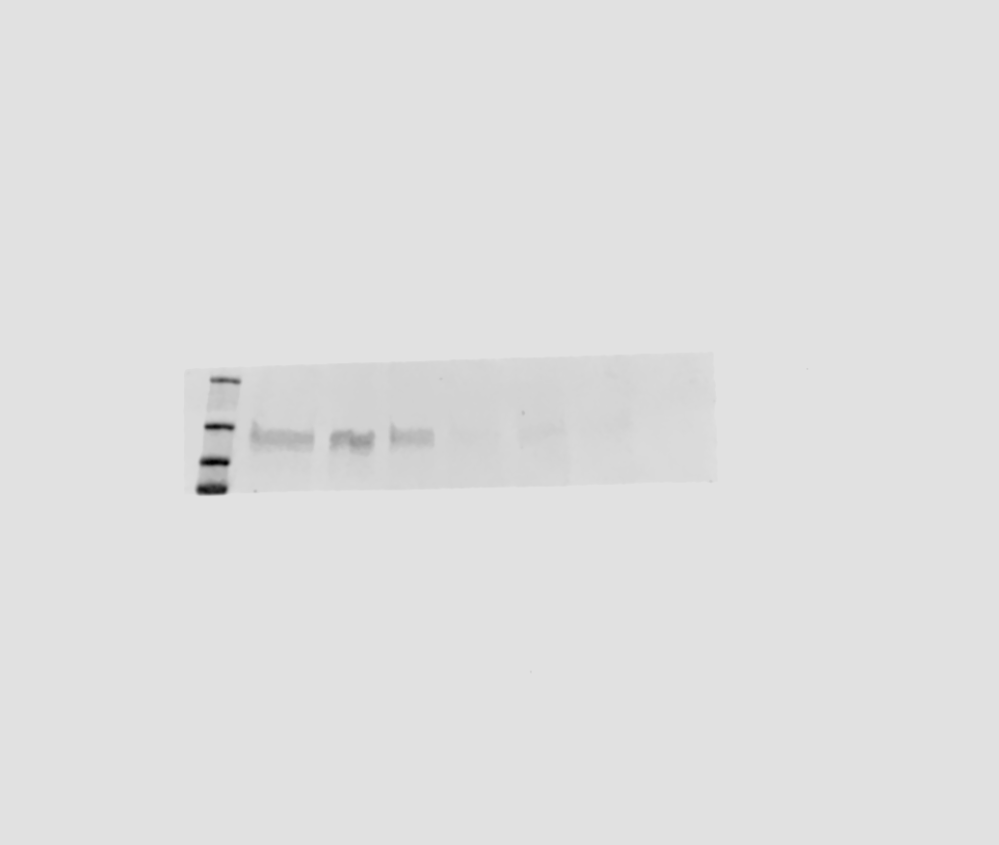

Supplement: Figure 6—source data 3. [file elife-75233-fig6-data3.zip › Figure 6 ΓÇô source data 3.tif]

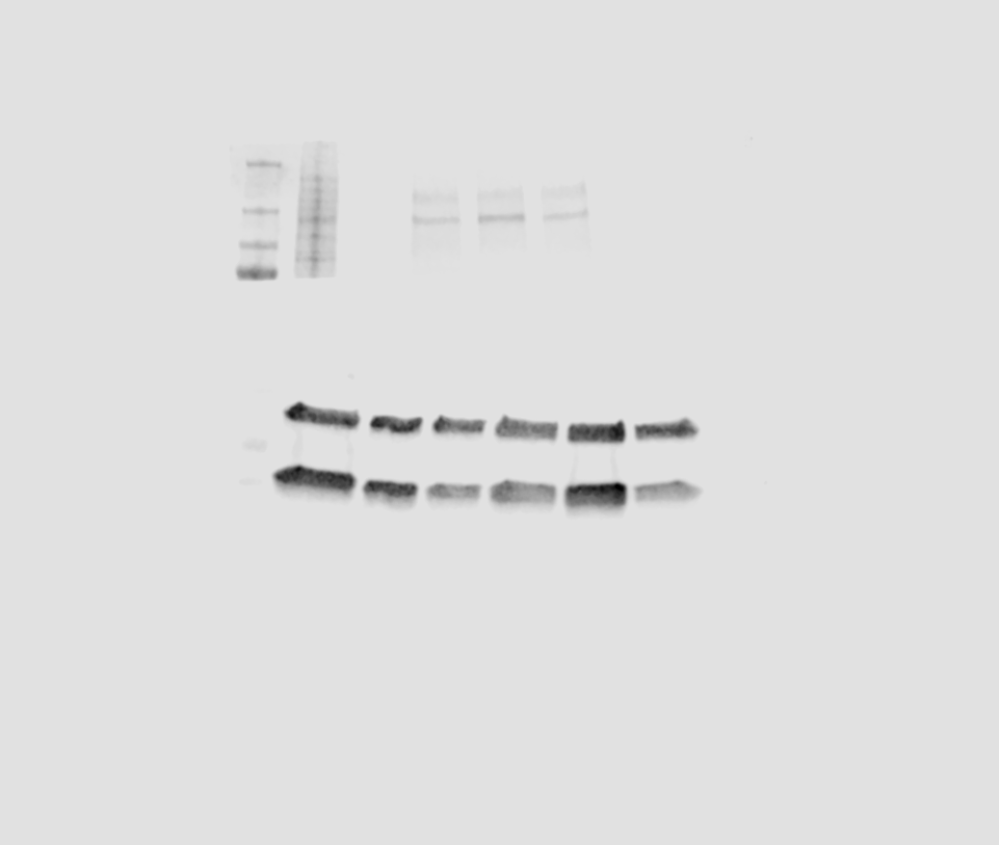

Supplement: Figure 6—source data 4. [file elife-75233-fig6-data4.zip › Figure 6 ΓÇô source data 4.tif]

**Cell Line: COS-7**

**Acc No : 87021302**

**Lot: 15I032**

**P +4**

**5th October 2015**

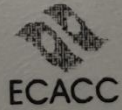

**ECACC**

**Cell Line: 293T**

**Acc No : 12022001**

**Lot: 16G020**

**p +6**

**2nd August 2016**

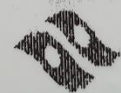

**ECACC**

Supplement: Supplementary file 2. — Labels of the vials of COS-7 and HEK-293T cell lines from ECACC purchased through Sigma-Aldrich in 2015 and 2016, respectively. [file elife-75233-supp2.pdf]
