# Supplementary material for: MDGAs are fast-diffusing molecules that delay excitatory synapse development by altering neuroligin behavior
Source: eLife. 2022 May 9;11:e75233. doi: 10.7554/eLife.75233 (PMC9084894; doi:10.7554/eLife.75233)
Supplement: Figure 2—figure supplement 1—source data 6. [file elife-75233-fig2-figsupp1-data6.pdf]

Figure 2 – figure supplement 1A

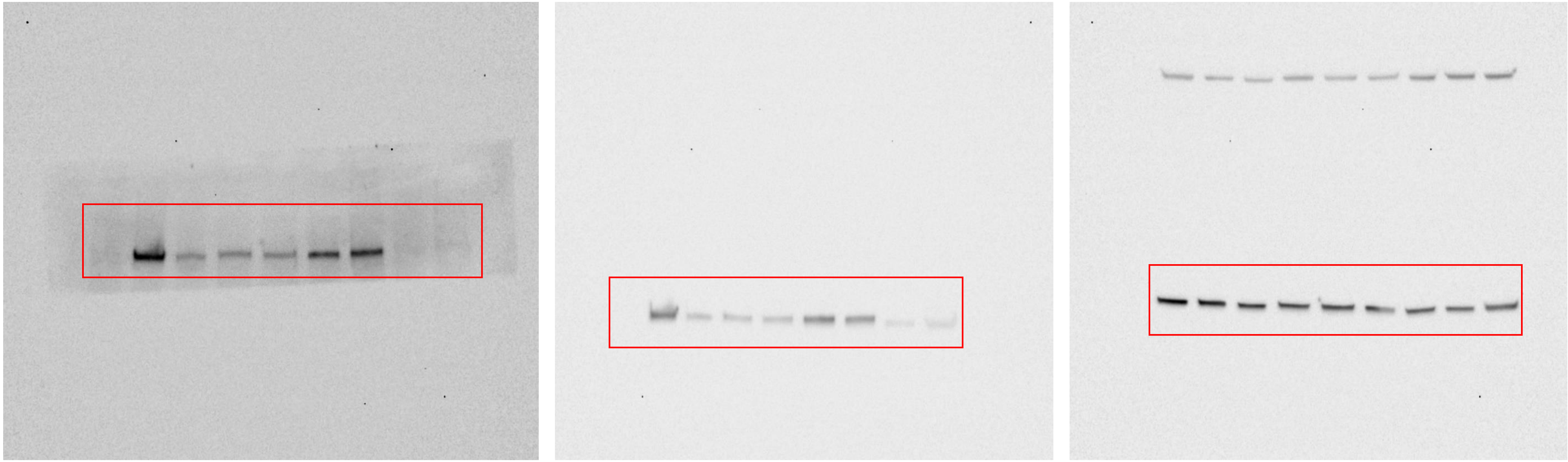

Anti-MDGA1 Anti-HA Anti-Tubulin

Figure 2 – figure supplement 1B

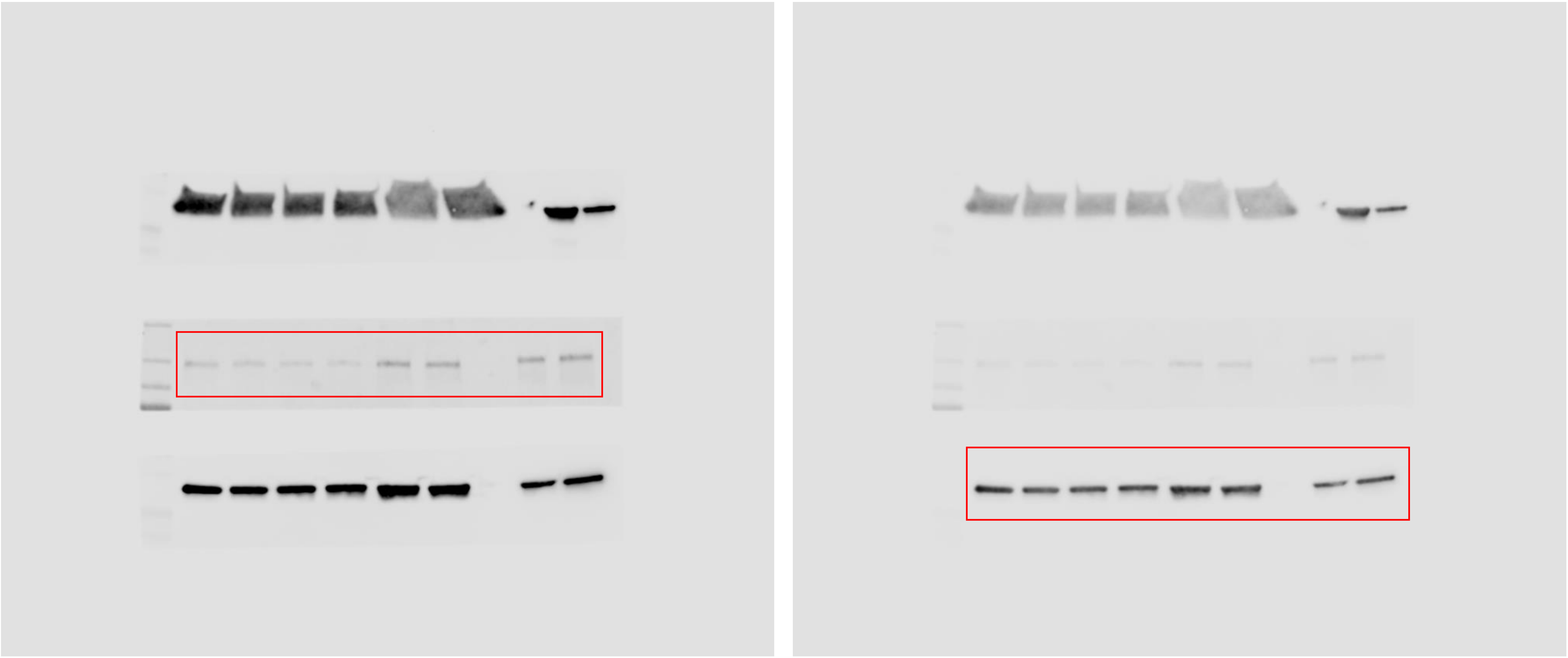

Anti-HA

Anti-Actin
