# Supplementary material for: MDGAs are fast-diffusing molecules that delay excitatory synapse development by altering neuroligin behavior
Source: eLife. 2022 May 9;11:e75233. doi: 10.7554/eLife.75233 (PMC9084894; doi:10.7554/eLife.75233)
Supplement: Figure 6—source data 5. [file elife-75233-fig6-data5.pdf]

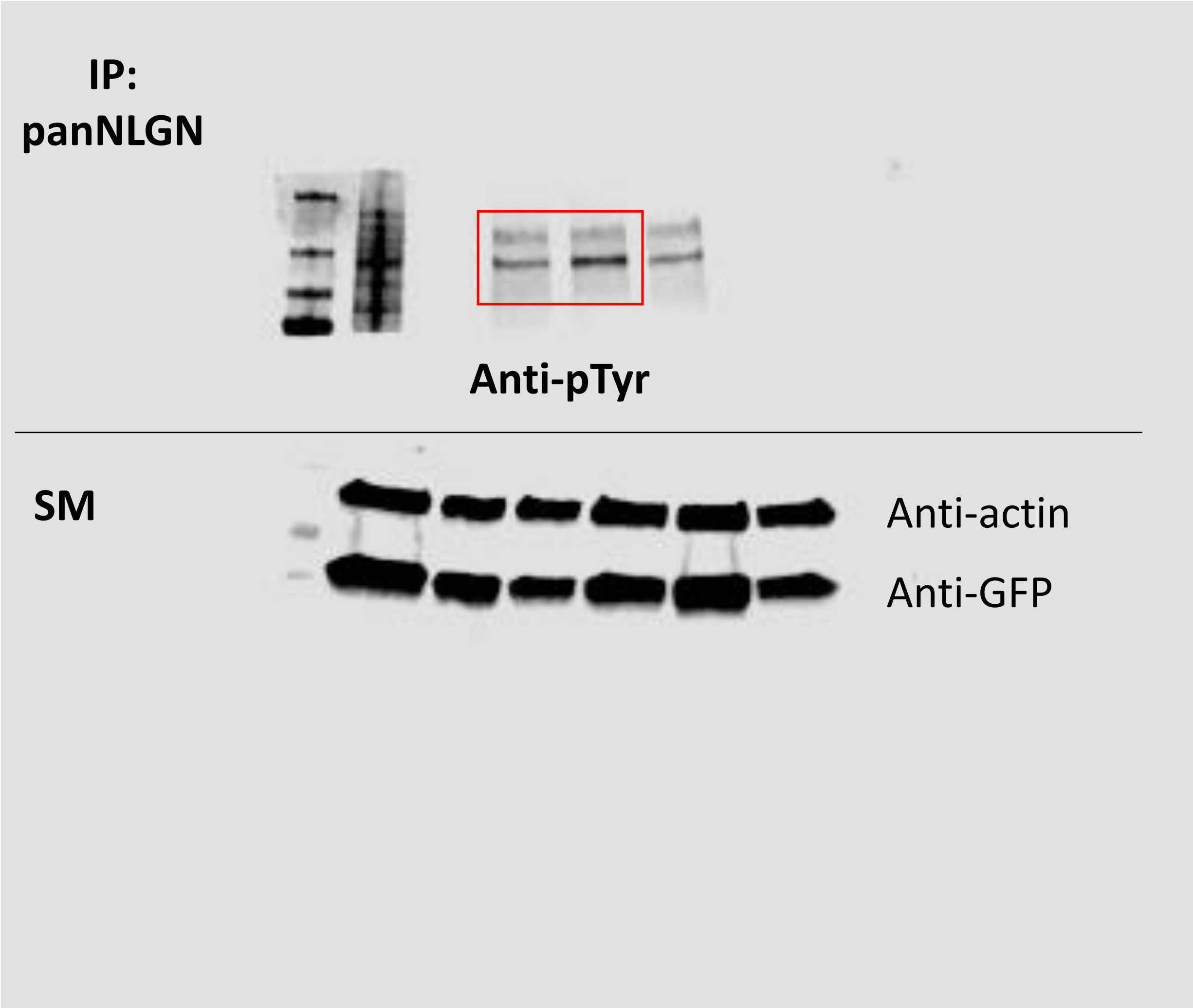
IP:
panNLGN
Anti-pTyr
SM
Anti-actin
Anti-GFP

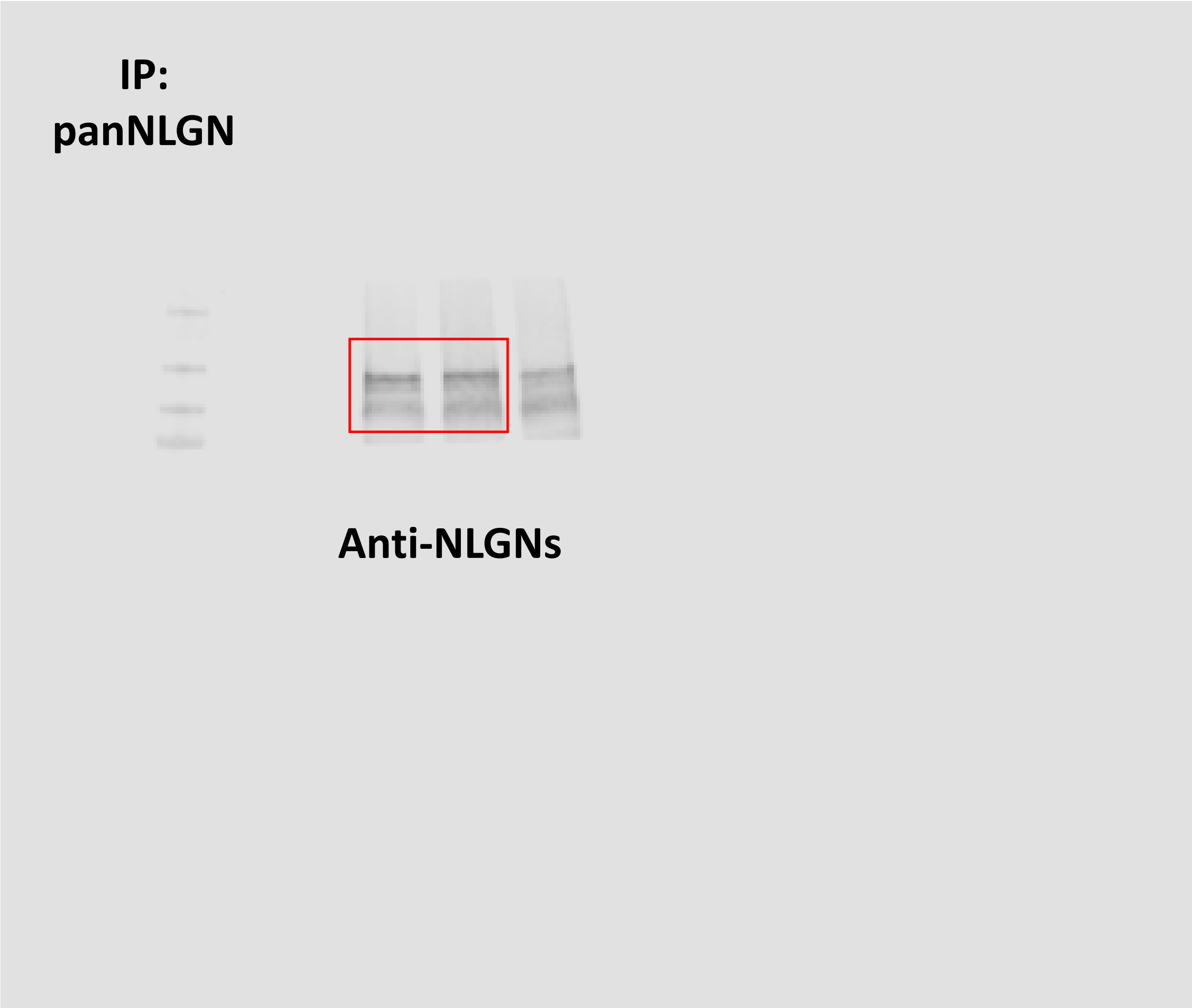
IP:
panNLGN
Anti-NLGNs

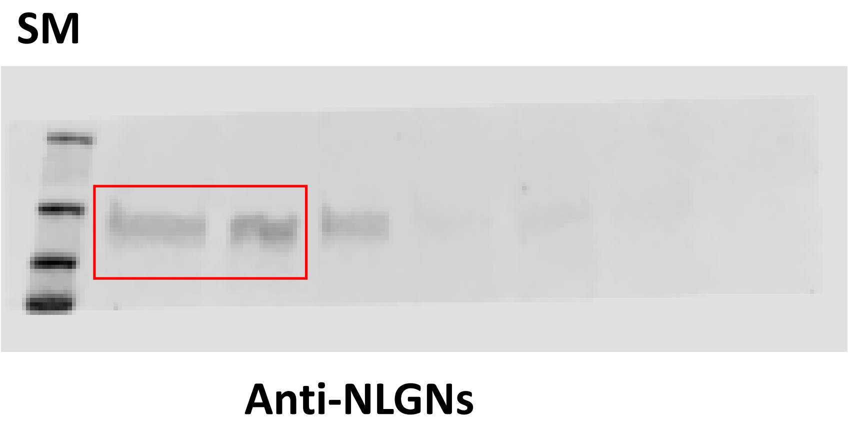
SM
Anti-NLGNs

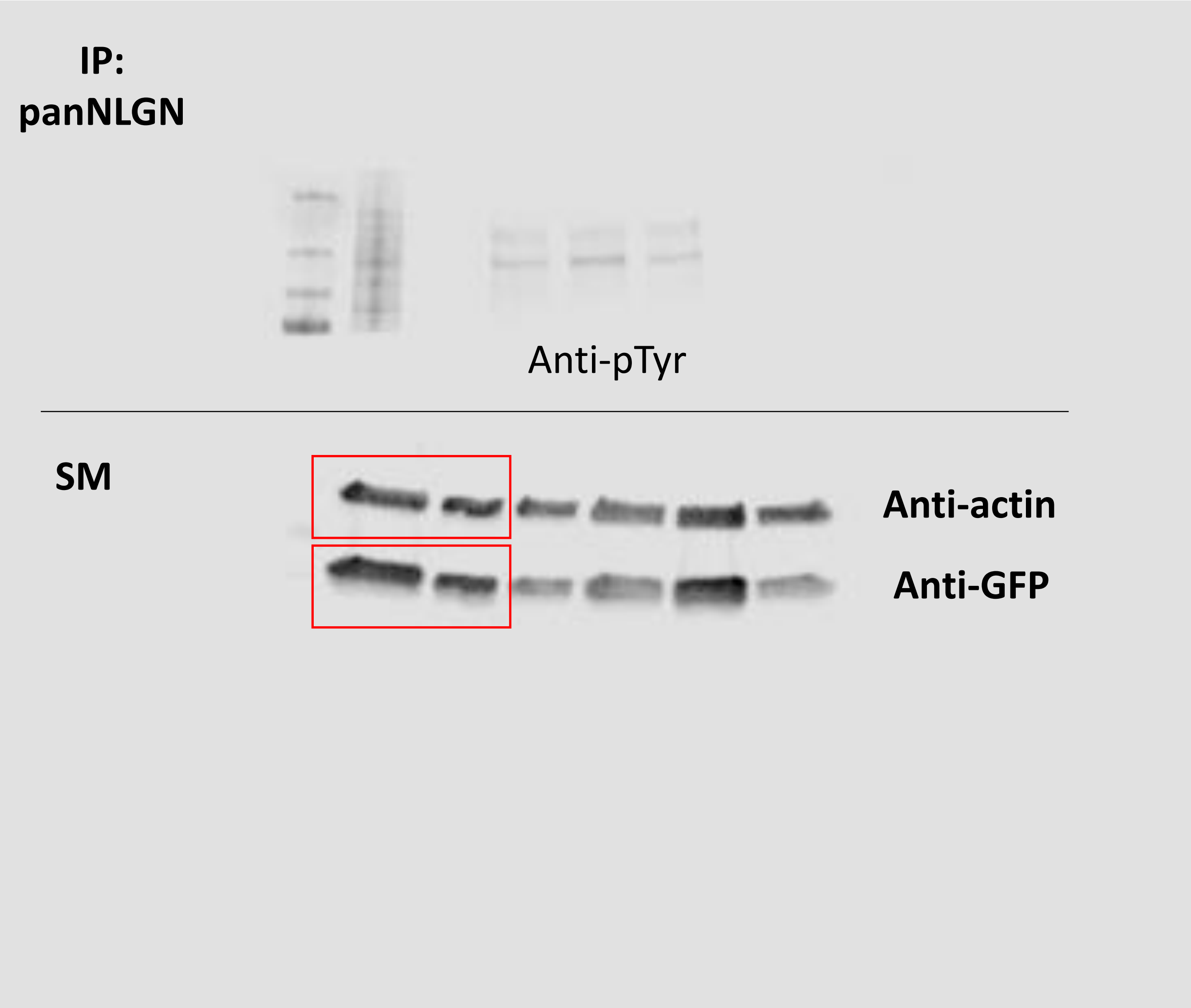
IP:
panNLGN
Anti-pTyr
SM
Anti-actin
Anti-GFP
