# Supplementary material for: MDGAs are fast-diffusing molecules that delay excitatory synapse development by altering neuroligin behavior
Source: eLife. 2022 May 9;11:e75233. doi: 10.7554/eLife.75233 (PMC9084894; doi:10.7554/eLife.75233)
Supplement: Supplementary file 1. — The number of experimental replicates ( = number of independent experiments performed), number of biological replicates ( = number of different cells analyzed), result of the normality test, statistical tests used to compare the data, and P-values associated to each test are given for each figure panel. [file elife-75233-supp1.docx]

**Supplementary file 1. Statistics table.**

| Figure | N (experimental replicates) | n  (biological replicates) | Normality test pass | Statistical test | p-value |
| --- | --- | --- | --- | --- | --- |
| 1 G | 3 | > 13 | No | ANOVA Kruskal Wallis;  Dunn´s multiple comparison test | ** 0.0078  **** 0.003  NS > 0.9999 |
| 1 H | 3 | > 13 | No | ANOVA Kruskal Wallis;  Dunn´s multiple comparison test | NS 0.1397  NS 0.0900  NS > 0.9999 |
| 1 J | 3 | > 25 | Yes | One way ANOVA | * 0.0183  ** 0.0068  NS 0.648 |
| 1 K | 3 | > 24 | Yes | One way ANOVA | ** 0.0024  *** 0.0003  NS 0.3312 |
| 2 B | 3 | > 5 | No | ANOVA Kruskal Wallis;  Dunn´s multiple comparison test | * 0.0301; 0.0372  ** 0.0013  NS > 0.9999 |
| 2 D | 3 | > 10 | No | ANOVA Kruskal Wallis;  Dunn´s multiple comparison test | * 0.0243  * 0.0134  *** 0.0006  NS > 0.9999 |
| 3 H | 3 | > 8 DIV 8 | No | ANOVA Kruskal Wallis;  Dunn´s multiple comparison test | MDGA1  **** < 0.0001 |
|  |  | > 12 DIV 10 |  |  |  |
|  |  | > 11 DIV 14 |  |  | MDGA2  **** < 0.0001 |
|  |  |  |  |  | NS > 0.9999 |
| 4 B | 3 | > 9 | No | ANOVA Kruskal Wallis;  Dunn´s multiple comparison test | *** 0.0069  *** 0.0012  NS 0.9025 |
| 4 D | 3 | > 5 | No | ANOVA Kruskal Wallis;  Dunn´s multiple comparison test | * 0.0355  NS > 0.9999  NS 0.1016 |
| 5 C | 3 | > 16 | No | ANOVA Kruskal Wallis;  Dunn´s multiple comparison test | NS 0.8059  NS > 0.999 |
| 5 F | 3 | > 8 | No | ANOVA Kruskal Wallis;  Dunn´s multiple comparison test | * 0.0265  NS 0.999  NS 0.7205 |
| 6 B | 3 | > 4 | No | ANOVA Kruskal Wallis;  Dunn´s multiple comparison test | NS 0.3185  NS > 0.9999  NS 0.2004 |
| 6 D | 3 | > 7 | No | ANOVA Kruskal Wallis;  Dunn´s multiple comparison test | NS 0.3394  NS 0.9999  NS 0.457 |
| 6 F | 7 | - | No | t-test Mann Whitney test | *** 0.0006 |
| 6 G | 7 | - | No | t-test Mann Whitney test | NS 0.4042 |
| 7 C | 3 | > 12 | Yes | Unpaired t test | ** 0.0030 |
| 7 G | 3 | > 12 | yes | Unpaired t test | ** 0.0026 |
| 8 B | 3 | > 17 | No | ANOVA Kruskal Wallis;  Dunn´s multiple comparison test | *** 0.0006  *** 0.0002  NS > 0.9999 |
| 8 C | 3 | > 17 | No | ANOVA Kruskal Wallis;  Dunn´s multiple comparison test | NS 0.0693  NS 0.6752  NS 0.0650 |
| 8 D | 3 | > 17 | No | ANOVA Kruskal Wallis;  Dunn´s multiple comparison test | NS 0.2199  ** < 0.0025  NS 0.9999 |
| 8 E | 3 | > 17 | No | ANOVA Kruskal Wallis;  Dunn´s multiple comparison test | NS 0.999 |
| 8 G | 5 | > 7 | No | ANOVA Kruskal Wallis;  Dunn´s multiple comparison test | NS 0.9999 |
| 8 H | 5 | > 7 | No | ANOVA Kruskal Wallis;  Dunn´s multiple comparison test | * 0.0374  ** 0.0055  NS > 0.9999 |
| 9 C | 3 | 5-15 | No | ANOVA Kruskal Wallis;  Dunn´s multiple comparison test | NS 0.1226  * 0.0349 |
| 9 D | 3 | 5-15 | No | ANOVA Kruskal Wallis;  Dunn´s multiple comparison test | NS > 0.9999  NS > 0.9999 |
| 9 E | 3 | 5-15 | No | Wilcoxon matched-pairs signed rank test;  Paired t-test | NS 0.8125  *** 0.0005  * 0.0342 |
| Supplemental |  |  |  |  |  |
| 1 – S1A | 3 | - | No | ANOVA Kruskal Wallis;  Dunn´s multiple comparison test | NS > 0.9999 |
| 1 – S1B | 3 | - | No | ANOVA Kruskal Wallis;  Dunn´s multiple comparison test | NS > 0.9999 |
| 1 – S1D | 3 | - | No | ANOVA Kruskal Wallis;  Dunn´s multiple comparison test | NS 0.8175 NLGN1  NS 0.225 NLGN2  NS 0.225 NLGN3  NS 0.175 gephyrin  * 0.0458 PSD-95  NS 0.0513 MDGA1  NS 0.225 GluA1 |
| 1 – S3B | 2 | > 58 | No | ANOVA Kruskal Wallis;  Dunn´s multiple comparison test | NS > 0.9999  NS 0.0677  NS 0.0511 |
| 1 – S3C | 2 | > 41 | Yes | One Way ANOVA | NS 0.0659  NS 0.1626  *** 0.0002 |
| 1 - S3D | 2 | > 41 | No | ANOVA Kruskal Wallis;  Dunn´s multiple comparison test | NS 0.9417  NS 0.3631  NS > 0.9999 |
| 1 - S3E | 2 | > 41 | No | ANOVA Kruskal Wallis;  Dunn´s multiple comparison test | NS 0.3047  NS 0.6689  NS > 0.999 |
| 2 - S1C | 2 | - | No | ANOVA Kruskal Wallis;  Dunn´s multiple comparison test | * 0.022  NS 0.0526  NS 0.0515  NS 0.791  NS 0.6504  NS > 0.9999  NS 0.7584 |
| 2 - S1D | 2 | - | No | ANOVA Kruskal Wallis;  Dunn´s multiple comparison test | NS 0.2782  ** 0.0024  *** 0.0007  NS > 0.999  NS 0.9678  NS > 0.9999  NS 0.3008 |
| 2 - S2C | 2 | > 42 | No | ANOVA Kruskal Wallis;  Dunn´s multiple comparison test | NS 0.894  **** < 0.0001 |
| 2 - S2D | 3 | > 53 | No | ANOVA Kruskal Wallis;  Dunn´s multiple comparison test | **** < 0.0001  ** 0.0058  NS 0.3384 |
| 2 - S2E | 4 | - | No | Unpaired t test | *** 0.0002 |
| 5 - S1D | 3 | > 81 DIV 8 | No | ANOVA Kruskal Wallis;  Dunn´s multiple comparison test | NS 0.0876  **** < 0.0001 |
|  |  | ­­> 12 DIV 10 |  |  | NS 0.3283  **** < 0.0001  NS 0.3899 |
|  |  | > 50 DIV 14 |  |  | *** 0.0006  ** 0.0051  NS > 0.9999 |
| 5 - S1E | 3 | > 81 DIV 8  > 12 DIV 10  > 58 DIV 14 | No | ANOVA Kruskal Wallis;  Dunn´s multiple comparison test | NS > 0.9999 |
| 6 – S1B | 2 | > 12 | No | t-test Mann Whitney test | NS 0.501 |
| 8 - S1C | 3 | > 13 | No | t-test Mann Whitney test | **** < 0.0001 |
| 8 - S1E | 2 | > 25 | No | Mann Whitney test | NS 0.95 |
| 8 - S1F | 2 | > 25 | No | Mann Whitney test | NS 0.88 |
| 8 – S2B | 1 | 5 | N/A | Chi-square | ** 0.01 |
| 8 – S2C | 1 | 5 | N/A | Chi-square | ** 0.01 |
| 8 – S2E | 1 | 5 | No | Pearson’s correlation test | *** < 0.001 |
| 8 – S2F | 1 | 5 | No | Mann-Whitney | NS [0.09-0.99] |
| 8 – S2G | 1 | 5 | No | Mann-Whitney | NS [0.18-0.99] |
| 8 – S3B | 3 | > 99 | No | ANOVA Kruskal Wallis;  Dunn´s multiple comparison test | NS 0.3686  NS > 0.999 |
| 8 – S3C | 3 | > 99 | No | ANOVA Kruskal Wallis;  Dunn´s multiple comparison test | NS 0.1904  NS 0.0663 |
| 8 – S3D | 3 | > 85 | No | ANOVA Kruskal Wallis;  Dunn´s multiple comparison test | ** 0.0096  NS 0.1564 |
| 8 – S3E | 3 | > 79 | No | ANOVA Kruskal Wallis;  Dunn´s multiple comparison test | **** < 0.0001 |
| 8 – S4B | 3 | > 33 | Yes | One way ANOVA | NS > 0.9999  NS 0.9533  NS > 0.9999 |
| 8 – S4C | 3 | > 33 | Yes | One way ANOVA | NS > 0.9999 |
| 8 – S4E | 3 | > 6 | No | ANOVA Kruskal Wallis;  Dunn´s multiple comparison test | NS > 0.9999 |
| 8 – S4F | 3 | > 6 | No | ANOVA Kruskal Wallis;  Dunn´s multiple comparison test | NS > 0.9999  NS 0.8584  NS > 0.9999 |
| 8 – S5B | 2 | > 9 | No | ANOVA Kruskal Wallis;  Dunn´s multiple comparison test | NS > 0.999  NS 0.6851  NS > 0.9999 |
| 8 – S5C | 2 | > 9 | No | ANOVA Kruskal Wallis;  Dunn´s multiple comparison test | NS > 0.9999  **** 0.0059  NS > 0.9999 |
| 8 – S5E | 2 | > 5 | No | ANOVA Kruskal Wallis;  Dunn´s multiple comparison test | NS 0.4464  NS 0.3373  NS > 0.999 |
| 8 – S5F | 2 | > 19 | No | ANOVA Kruskal Wallis;  Dunn´s multiple comparison test | NS 0.3725  **** < 0.0001  NS > 0.9999 |
| 9 - S1A | 3 | 5-13 | Yes/No | Wilcoxon matched-pairs signed rank test  Paired t-test | NS 0.8125  * 0.0171  * 0.0335 |
| 9 - S1B | 3 | 5-13 | Yes/No | Wilcoxon matched-pairs signed rank test  Paired t-test | NS 0.625  NS 0.1677  NS 0.6195 |
| 9 - S1D | 3 | 5-29 | No | ANOVA Kruskal Wallis;  Dunn´s multiple comparison test | NS > 0.9999 |
